# Supplementary material for: Deep genomic characterization highlights complexities and prognostic markers of pediatric acute myeloid leukemia
Source: Commun Biol. 2023 Mar 31;6:356. doi: 10.1038/s42003-023-04732-2 (PMC10066286; doi:10.1038/s42003-023-04732-2)
Supplement: Supplementary file 2 — Supplementary Information [file 42003_2023_4732_MOESM2_ESM.pdf]

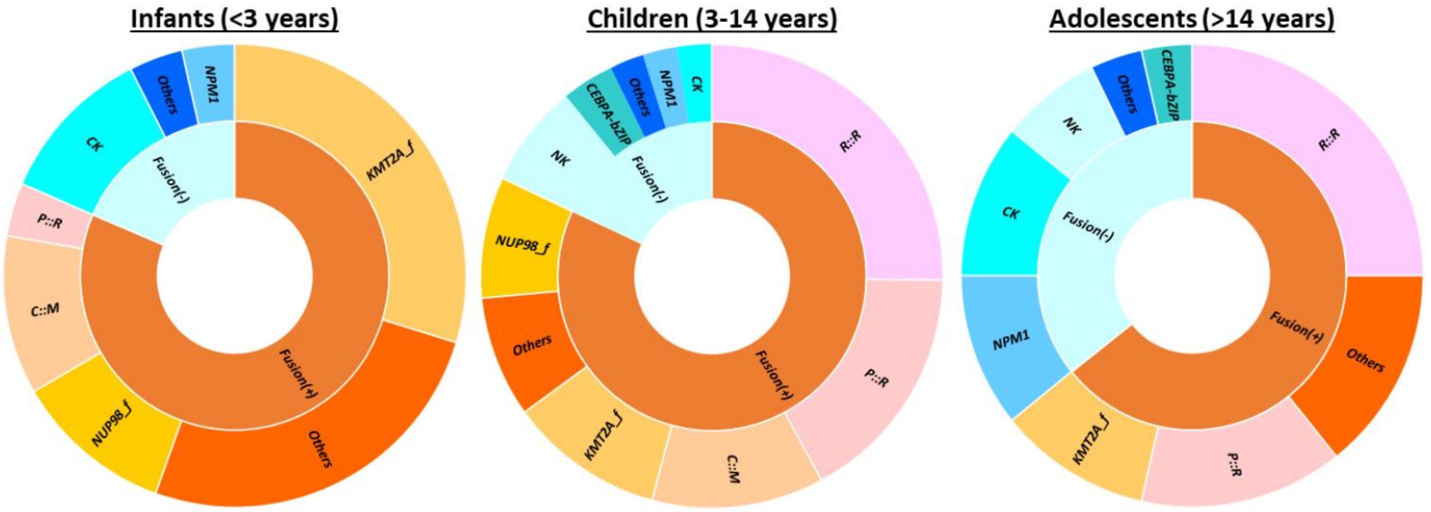

	Molecular/cytogenetic features	No. of infants (*n*=27)	%	No. of children (*n*=83)	%	No. of adolescents (*n*=28)	%	*P*-value	Adjusted *P*-value
Fusion(-)	CK	3	11%	2	2%	3	11%	0.062	0.347
	CEBPA-bZIP	0	0%	3	4%	1	4%	0.822	0.904
	NPM1	1	4%	2	2%	3	11%	0.186	0.347
	NK	0	0%	6	7%	2	7%	0.463	0.566
	Others	1	4%	2	2%	1	4%	1.000	1.000
Fusion(+)	*R::R*	0	0%	21	25%	7	25%	0.004	**0.047**
	P::R	1	4%	14	17%	4	14%	0.252	0.347
	C::M	3	11%	10	12%	0	0%	0.133	0.347
	*KMT2A*_f	8	30%	9	11%	3	11%	0.061	0.338
	*NUP98*_f	3	11%	7	8%	0	0%	0.174	0.347
	Others	7	26%	7	8%	4	14%	0.065	0.347

Supplementary Fig. 1. Distribution of FGs across age groups. Pediatric AML patients were stratified into three age groups (infants, children and adolescents) as previously described in the TARGET-AML study.[1] Only the 138 patients with suitable materials for FG studies were included in this analysis. The table below shows the number of patients with different molecular/cytogenetic features in each age group. *P*-values were calculated by the Fisher's exact test and corrected for multiple comparisons using the Benjamini-Hochberg method. CK, complex karyotype; *CEBPA*-bZIP, *CEBPA*-basic leucine zipper; NK, normal karyotype; *R::R*, *RUNX1::RUNX1T1*; *P::R*, *PML::RARA*; *C::M*, *CBFB::MYH11*; *KMT2A*_f, *KMT2A* fusions; *NUP98*_f, *NUP98* fusions.

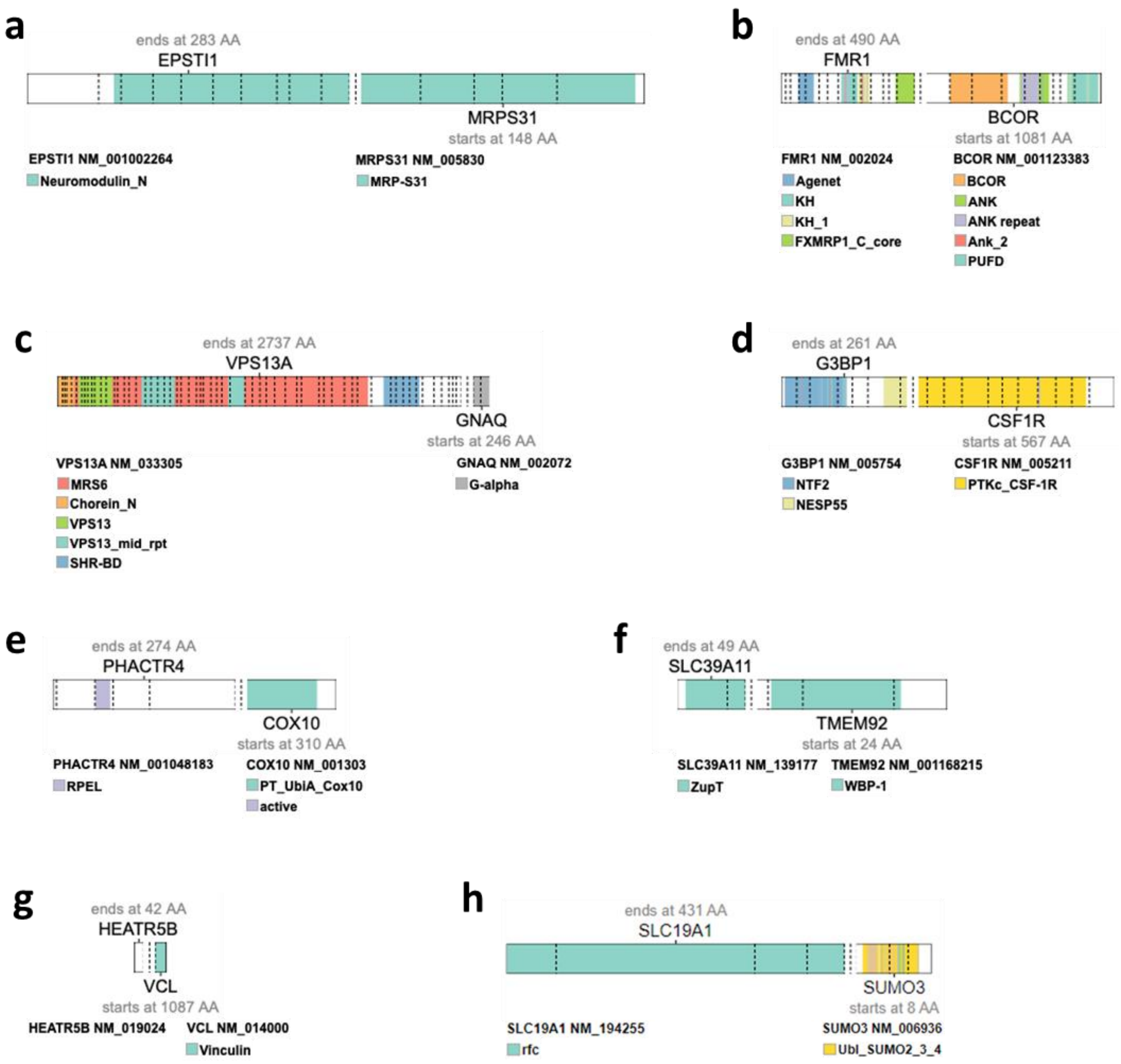

Supplementary Fig. 2. Novel in-frame FGs identified in this study. Schematic diagrams of FGs generated by ProteinPaint.[2] **a** EPSTI1::MRPS31. **b** FMR1::BCOR. **c** VPS13A::GNAQ. **d** G3BP1::CSF1R. **e** PHACTR4::COX10. **f** SLC39A11::TMEM92. **g** HEATR5B::VCL. **h** SLC19A1::SUMO3. Protein domains present in the fusion proteins are shown. The novel STIM1::MXD3, STIM1::F12 and RUNX1::ERG are shown in Fig. 1d and 2a.

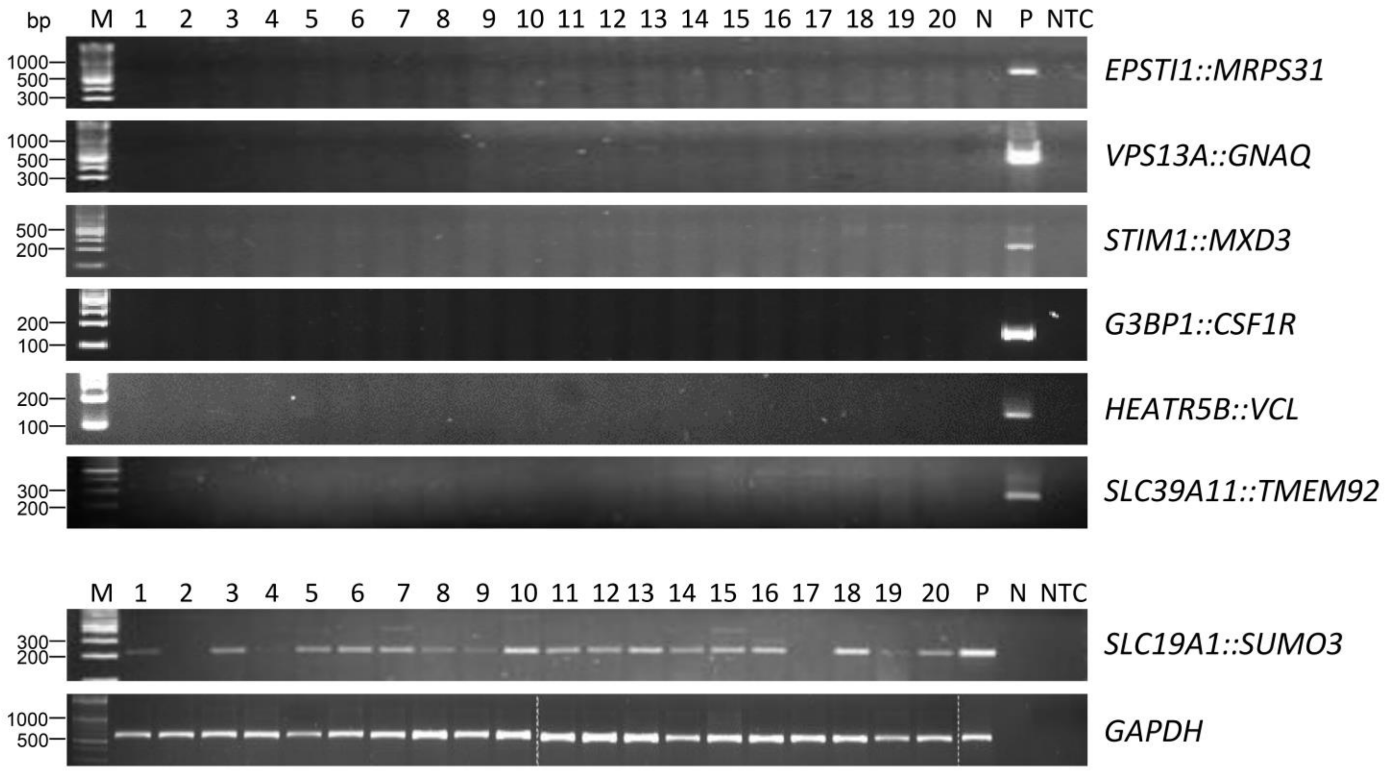

Supplementary Fig. 3. Expression of novel FGs in normocellular bone marrow samples. Representative RT-PCR results of seven novel FGs in 20 normocellular bone marrow samples (1-20) from individuals without a prior hematological malignancy. *GAPDH* served as the internal control. Dotted lines indicate merged lanes from the same gel. Lanes were merged to show the 20 bone marrow samples with adequate RNA quality as indicated by noticeable expression of *GAPDH*. M, size marker; N, no reverse transcriptase control; P, the patient sample positive for the FG; NTC, no template control.

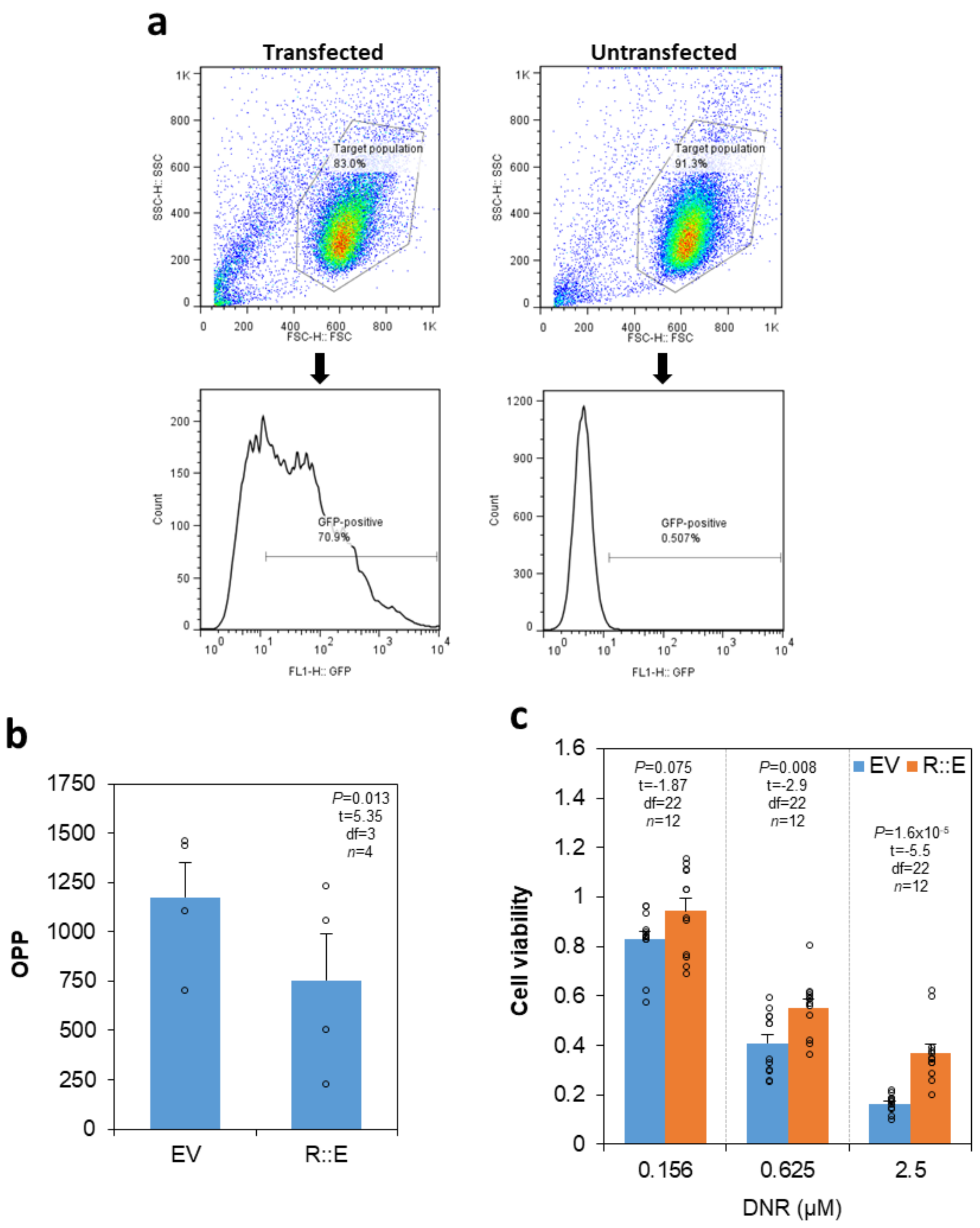

Supplementary Fig. 4. Overexpression of RUNX1::ERG inhibited protein synthesis and conferred chemotherapy resistance. K562 cells were transfected with LeGO-iG2-RUNX1::ERG (R::E) or the empty LeGO-iG2 (EV). **a** Gating strategy for flow cytometric analysis of transfected K562 cells. Target cell population was first gated, and green fluorescent protein (GFP)-positive cells were identified by parallel analysis of untransfected cells. The GFP-positive (transfected) cells were then analysed for the desired functional properties. **b** Incorporation of O-propargyl-puromycin (OPP) (median fluorescence intensity) into newly translated proteins in the transfected cells was measured after 72 hours of the transfection. **c** After 72 hours of transfection, cells were treated with the indicated concentrations of daunorubicin (DNR) for 48 hours and viability was measured with the CellTiter-Glo assay. Cell viability was relative to the vehicle-treated control. In panels **b** and **c**, data are expressed as mean ± SE from four independent experiments. The number of values used to calculate the statistics (*t* test) in each group is indicated.

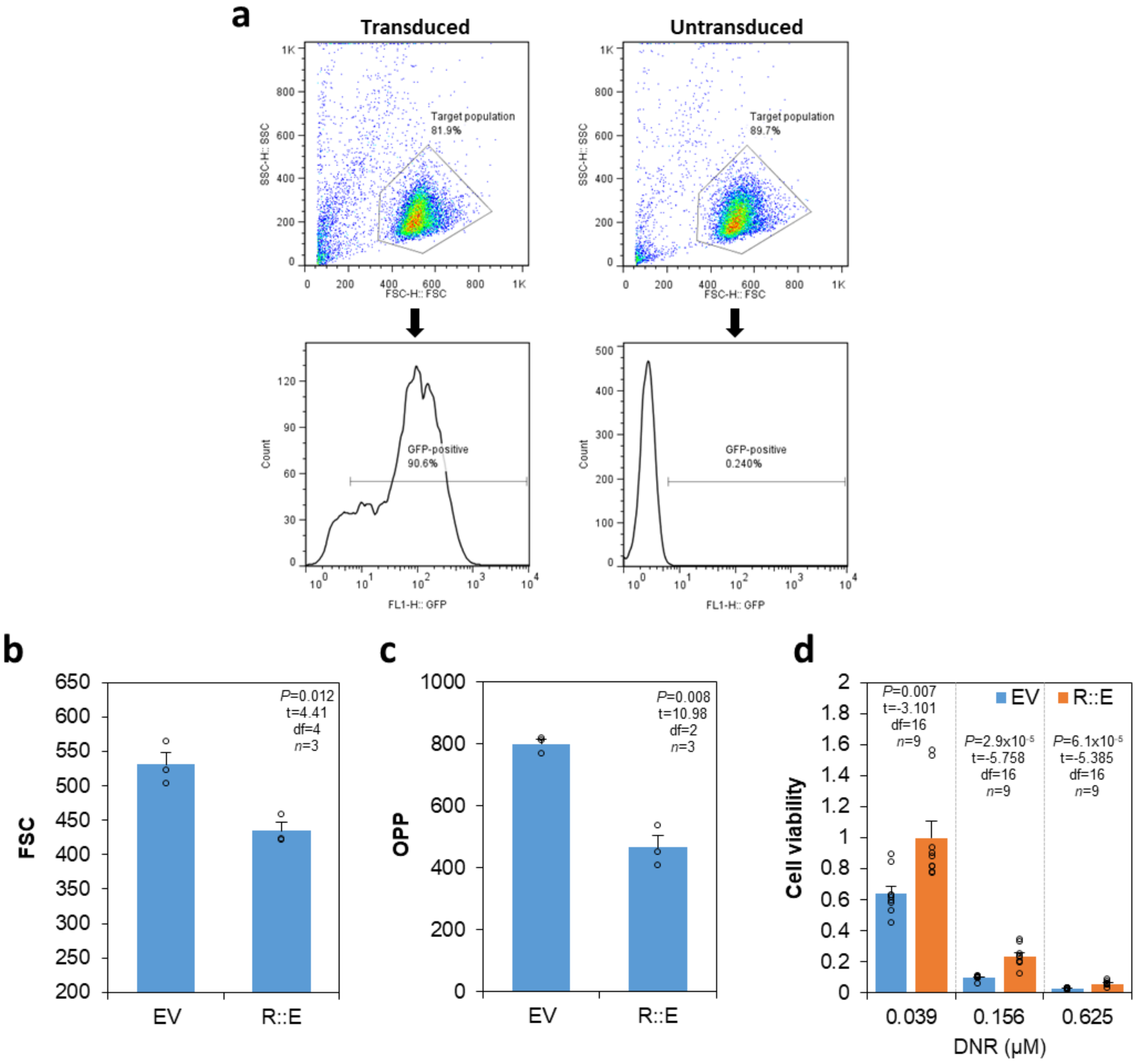

Supplementary Fig. 5. Effects of RUNX1::ERG overexpression on U937 myeloid leukemia cells. U937 cells were transduced with RUNX1::ERG (R::E) or the empty vector (EV) lentivirus at a multiplicity of infection of 4. **a** Gating strategy for flow cytometric analysis of transduced U937 cells. Target cell population was first gated, and green fluorescent protein (GFP)-positive cells were identified by parallel analysis of untransduced cells. The GFP-positive (transduced) cells were then analysed for the desired functional properties. Cell size (**b**) and incorporation of O-propargyl-puromycin (OPP) into newly translated proteins (**c**) in the transduced cells were measured after 96 hours of the transduction. The median fluorescence intensity of forward size scatter (FSC) and OPP signal are shown. **d** After 96 hours of transduction, cells were treated with the indicated concentrations of daunorubicin (DNR) for 48 hours and viability was measured with the CellTiter-Glo assay. Cell viability was relative to the vehicle-treated control. In panels **b-d**, data are expressed as mean ± SE from three independent experiments. The number of values used to calculate the statistics (*t* test) in each group is indicated.

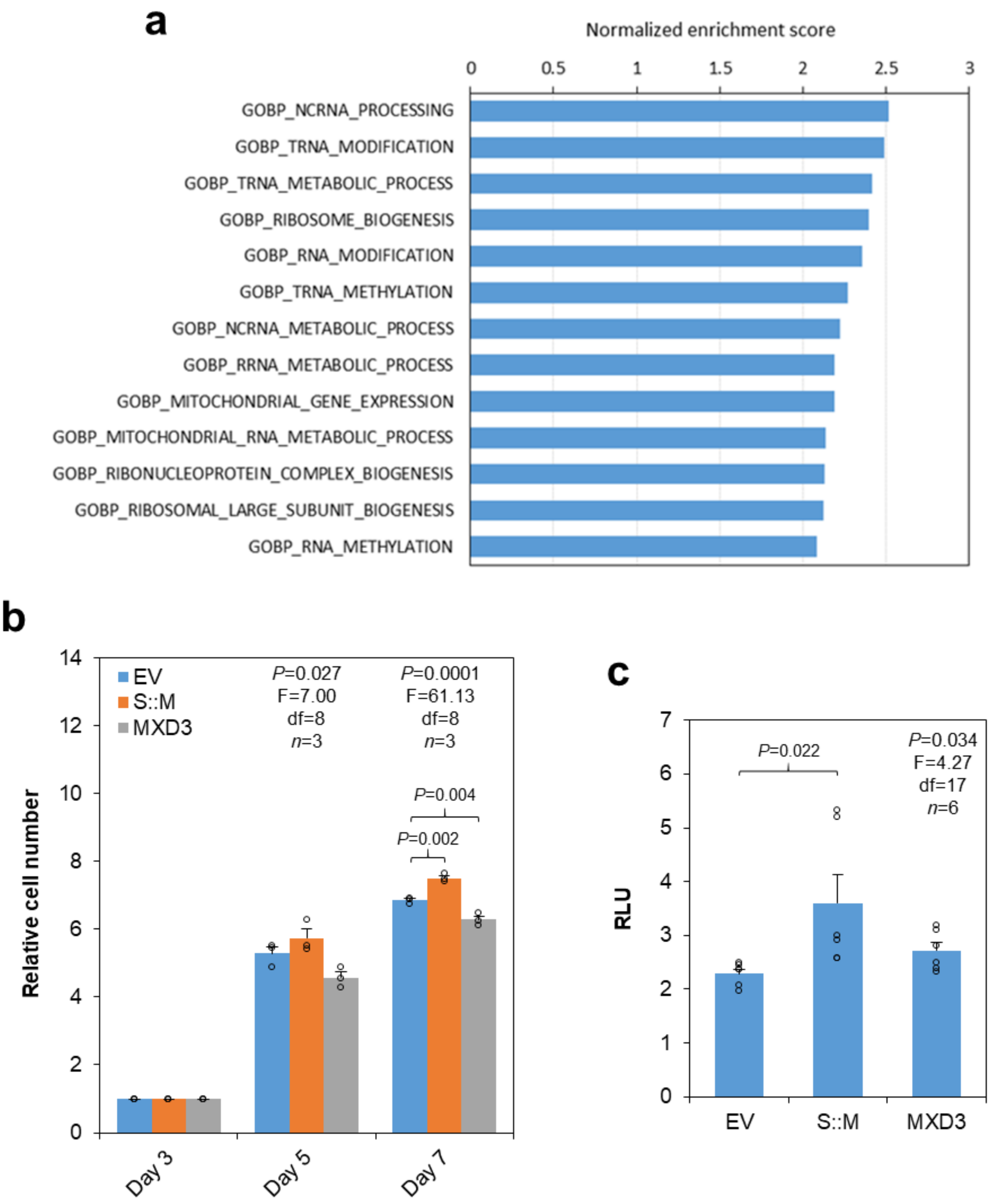

Supplementary Fig. 6. Differential biological properties of STIM1::MXD3. a GSEA comparing K562 cells transfected with pCMV-HA plasmid expressing STIM1::MXD3, MXD3 or the empty vector (EV). Two independent transfections and transcriptome sequencing were performed for each of the group. Pre-ranked gene lists were generated by comparing STIM1::MXD3 or MXD3 with the EV group, and the Gene Ontology biological process gene sets were analysed. Gene sets that were significantly (FDR<0.05 and FWER-adjusted *P*-value<0.05) associated with STIM1::MXD3 but not MXD3 are shown. **b** Effects of STIM1::MXD3 (S::M) and MXD3 overexpression on K562 cell proliferation as assessed by trypan blue cell counting. Cell number was relative to the 72-hour post-transfection time point. **c** Effects of STIM1::MXD3 and MXD3 overexpression on K562 cell viability as determined by CellTiter-Glo assays. Luminescence signal was measured 7 days post-transfection and relative to the 72-hour post-transfection time point. RLU, relative luminescence. Data in **b** and **c** are expressed as mean ± SE from 3 independent experiments. The number of values used to calculate the statistics (One-way ANOVA followed by Dunnett's test) in each group is indicated.

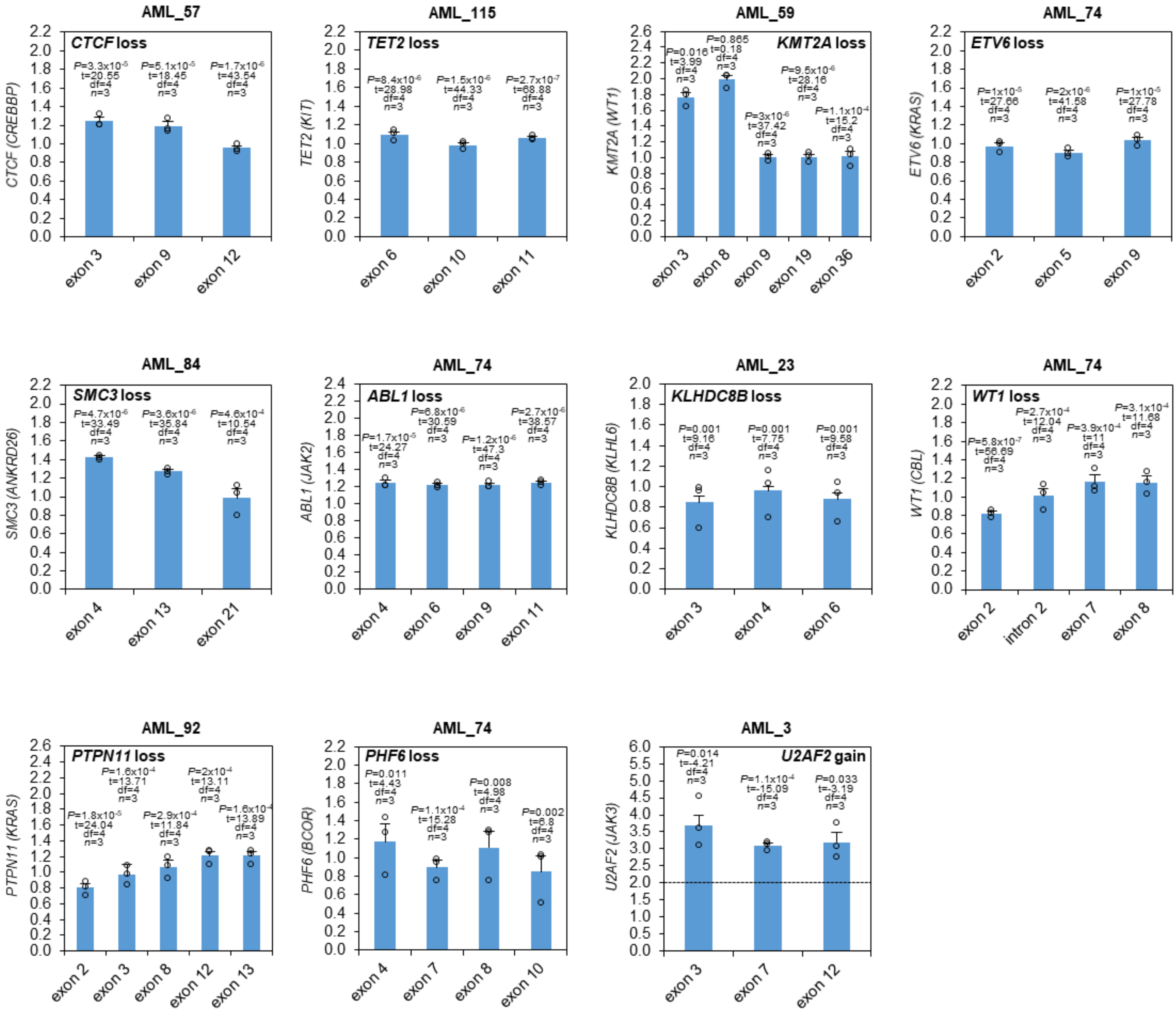

Supplementary Fig. 7. Validation of copy number alterations by qPCR. At least three different genomic regions of the involved genes were analysed and the results were normalized to another gene region (*bracketed*) of the same chromosome without copy number changes. Gene copy numbers were compared to those obtained from normal peripheral blood samples (*n*=3) used in quandico/CNVkit analysis. All qPCR measurements were performed in triplicates. Data are expressed as mean ± SE and the *t* test was used for statistical analysis. Representative results are shown.

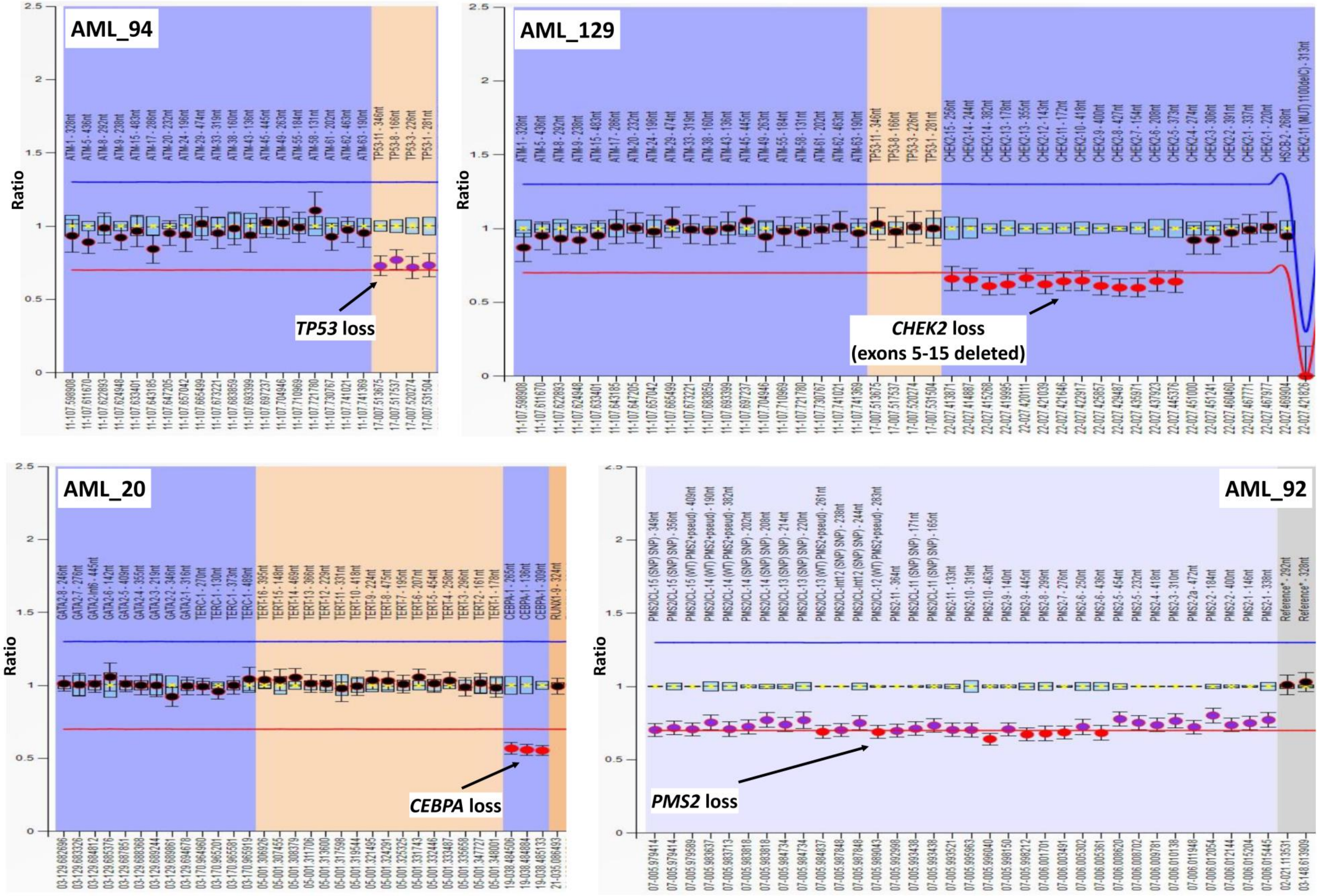

Supplementary Fig. 8 continued

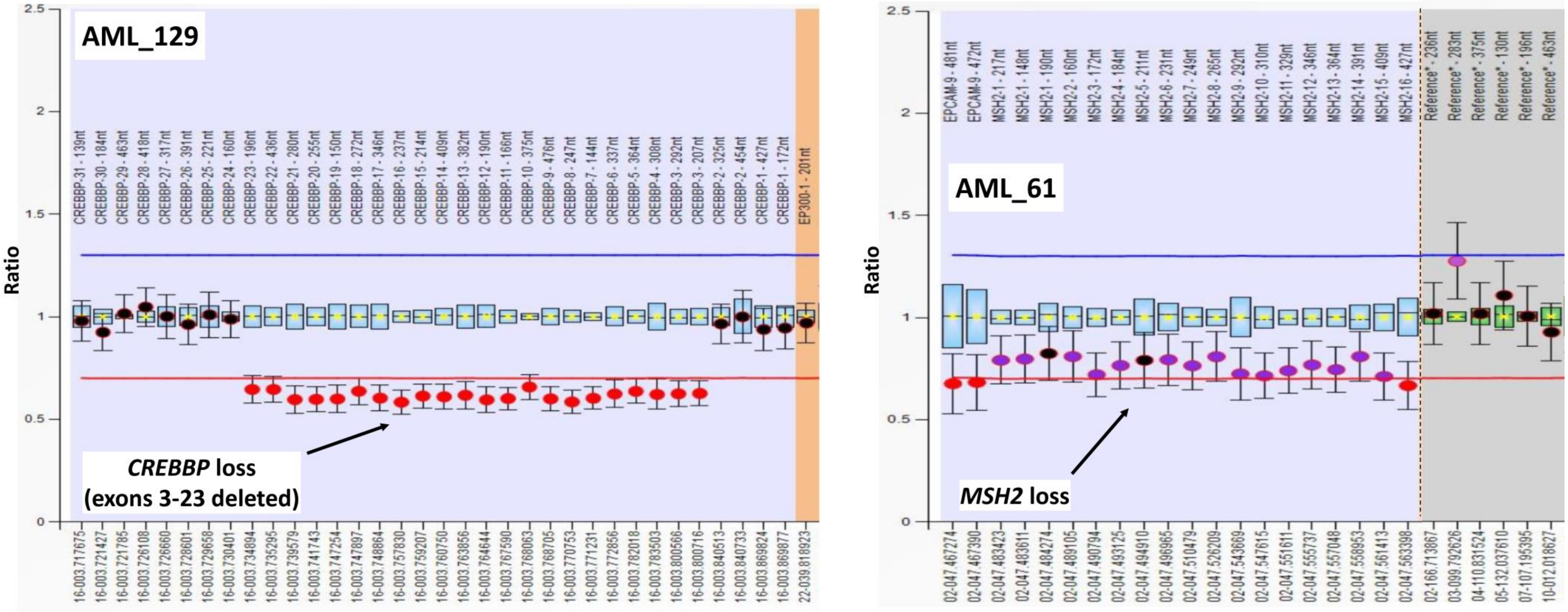

Supplementary Fig. 8. Validation of copy number alterations by MLPA. Representative MLPA results showing focal gene deletions in different patient samples. Exon numbers and the normalized probe ratios are shown above the charts and on the y-axis, respectively. Red dots indicate losses, while purple dots indicate significantly decreased signals compared to the reference control samples (*n*=3). Black dots indicate no copy number changes. Error bars indicate the 95% confidence intervals of the probes' ratio.

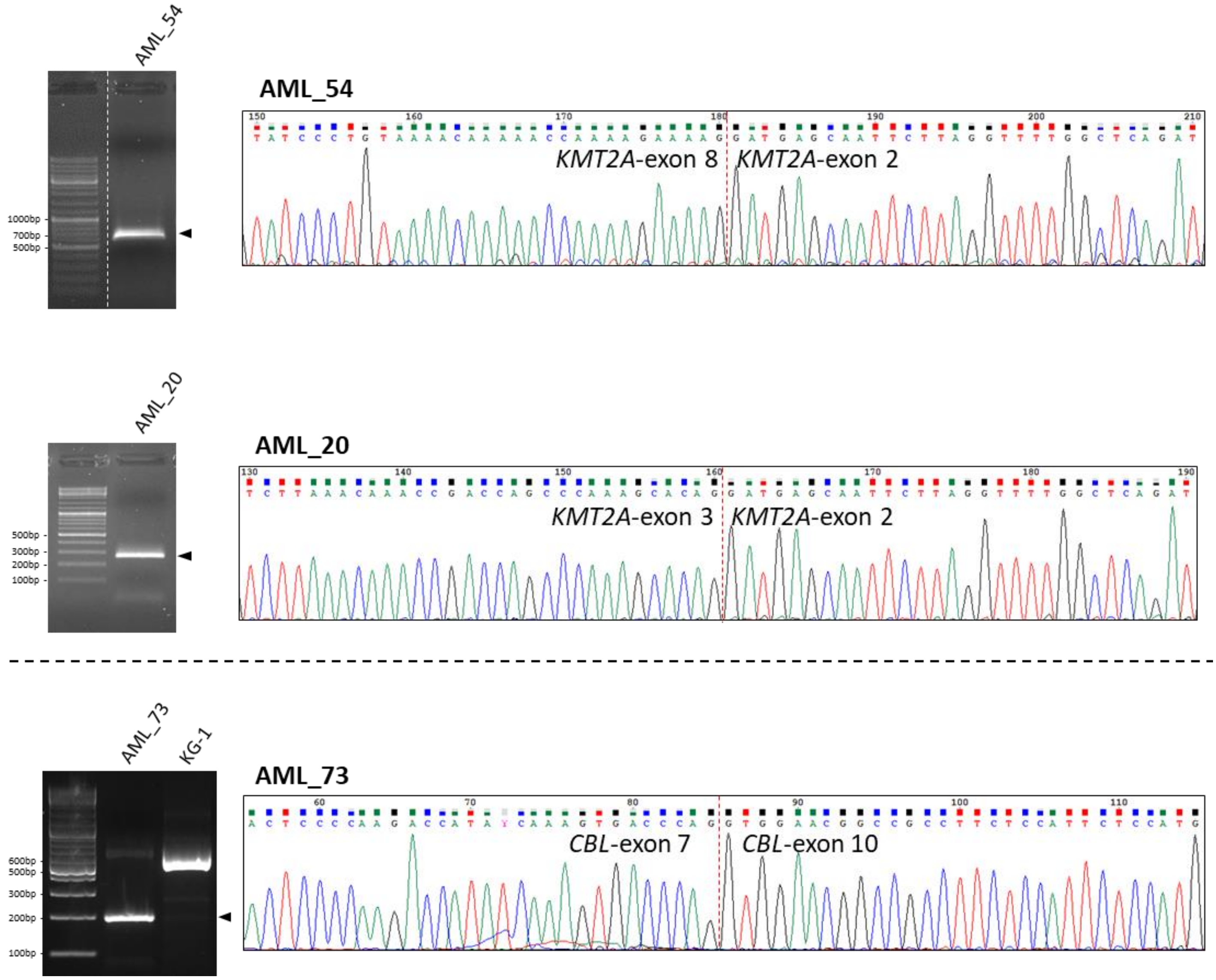

Supplementary Fig. 9. Validation of *KMT2A* partial tandem duplications (PTDs) and *CBL* intragenic deletion. *KMT2A*-PTDs and *CBL* deletion were validated by RT-PCR, followed by Sanger sequencing. A novel PTD involving only exons 2-3 was identified in AML_20. Deletion of *CBL* exons 8-9 was detected in AML_73 but not the negative control KG-1 cell line (wild-type PCR product of 532 bp). Arrowheads indicate the mutant PCR products. The white dotted line indicates merged lanes from the same gel. Lanes were merged to show the patient sample (AML_54) among others with *KMT2A*-PTD.

Functional pathways	Mutated genes
**Signaling**	*BCR, BRAF, CBL, CBLB, CSF1R, CSF3R, DNM2, EGFR, FLT3, JAK2, JAK3, KDR, KIT, KRAS, MPL, NF1, NOTCH1, NRAS, NTRK3, P2RY2, PDGFRA, PTPN11, STAT3*
**Transcription factor**	*BCL6, CEBPA, CUX1, ETV6, GATA1, GATA2, IKZF1, MYC, RUNX1*
**Chromatin regulation**	*ASXL1, ASXL2, BCOR, BCORL1, CREBBP, DAXX, EZH2, KAT6A, KDM6A, KMT2C, PHF6, SETBP1, SUZ12*
**DNA methylation**	*DNMT3A, IDH1, IDH2, TET2, WT1*
NPM1	*NPM1*
**Cohesin**	*CTCF, RAD21, SMC1A, SMC3, STAG2*
**Tumor suppressor**	*CDKN2A, RB1, TP53*
**Splicing**	*DDX41, PRPF8, SF1, SF3A1, SF3B1, U2AF1*
**DNA repair**	*ATM, BRCA1, BRCA2, MLH1, MSH2, PMS2, WRN*
**Adhesion**	*GJB3, PCDHB1, RELN*
**Others**	*ANKRD26, HNRNPK, SRP72, TERT, XPO1*

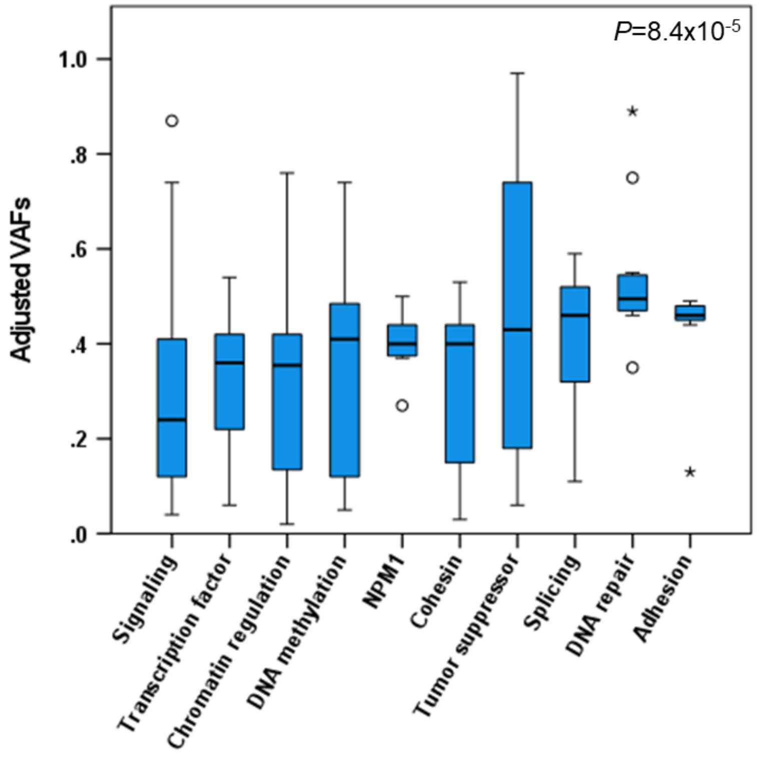

Supplementary Fig. 10. Variant allele frequencies (VAFs) of genes grouped into different pathways. *Top*, Mutated genes were grouped into distinct functional pathways. *Bottom*, A boxplot showing the VAFs of genes in different categories in the entire cohort of 147 patients. VAFs were adjusted for local copy number determined by quandico/CNVkit. The boxes represent the lower and upper quartiles, with the lines inside representing the median. The whiskers represent the maximum and minimum excluding outliers and extreme values. Small circles and asterisks represent outliers (>1.5 × interquartile range) and extreme values (>3 × interquartile range), respectively. *P*-value was calculated by the Kruskal-Wallis test.

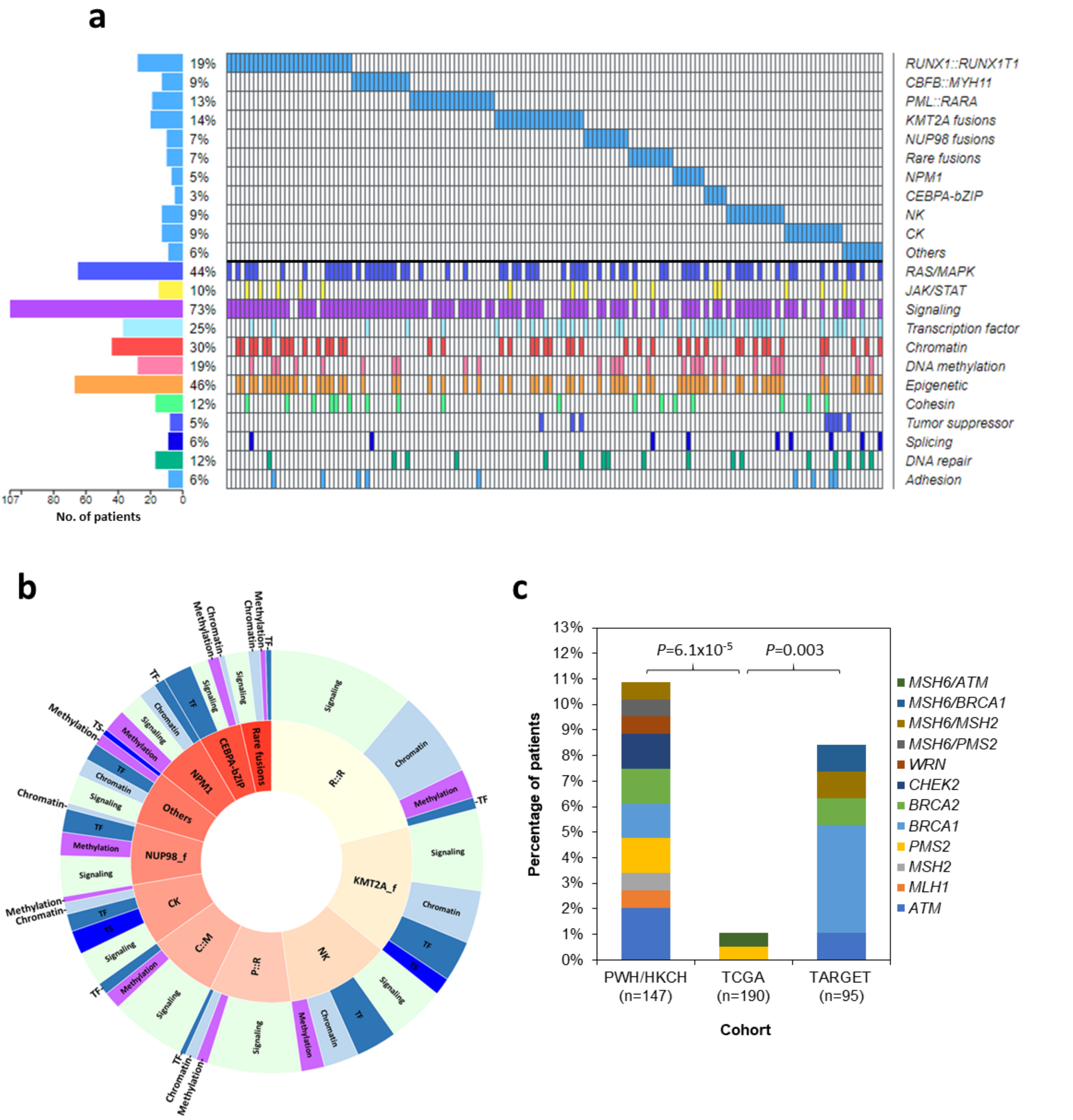

Supplementary Fig. 11. Mutational spectrum in pediatric AML. a A waterfall plot showing the distribution of pathway alterations among different molecular/cytogenetic subtypes. Pathways are shown on the right of the plot, while the number/percentage of the patients with the alterations are shown on the left. **b** A sunburst chart showing the distribution of alterations of 5 pathways (signaling, transcription factor (TF), chromatin regulation, DNA methylation and tumor suppressor (TS)) (the outer ring) that were differentially associated with disease subtypes (the inner ring). **c** Alterations of DNA repair genes among pediatric (PWH/HKCH and TARGET) and adult (TCGA) AML patient cohorts. Putative driver alterations including small mutations and gene deletions were included in the comparison. Pathogenicity of small mutations was evaluated as described in the Methods. Data for the TARGET- and TCGA-AML cohorts were obtained from cBioportal. *P*-values were calculated by the Fisher's exact test.

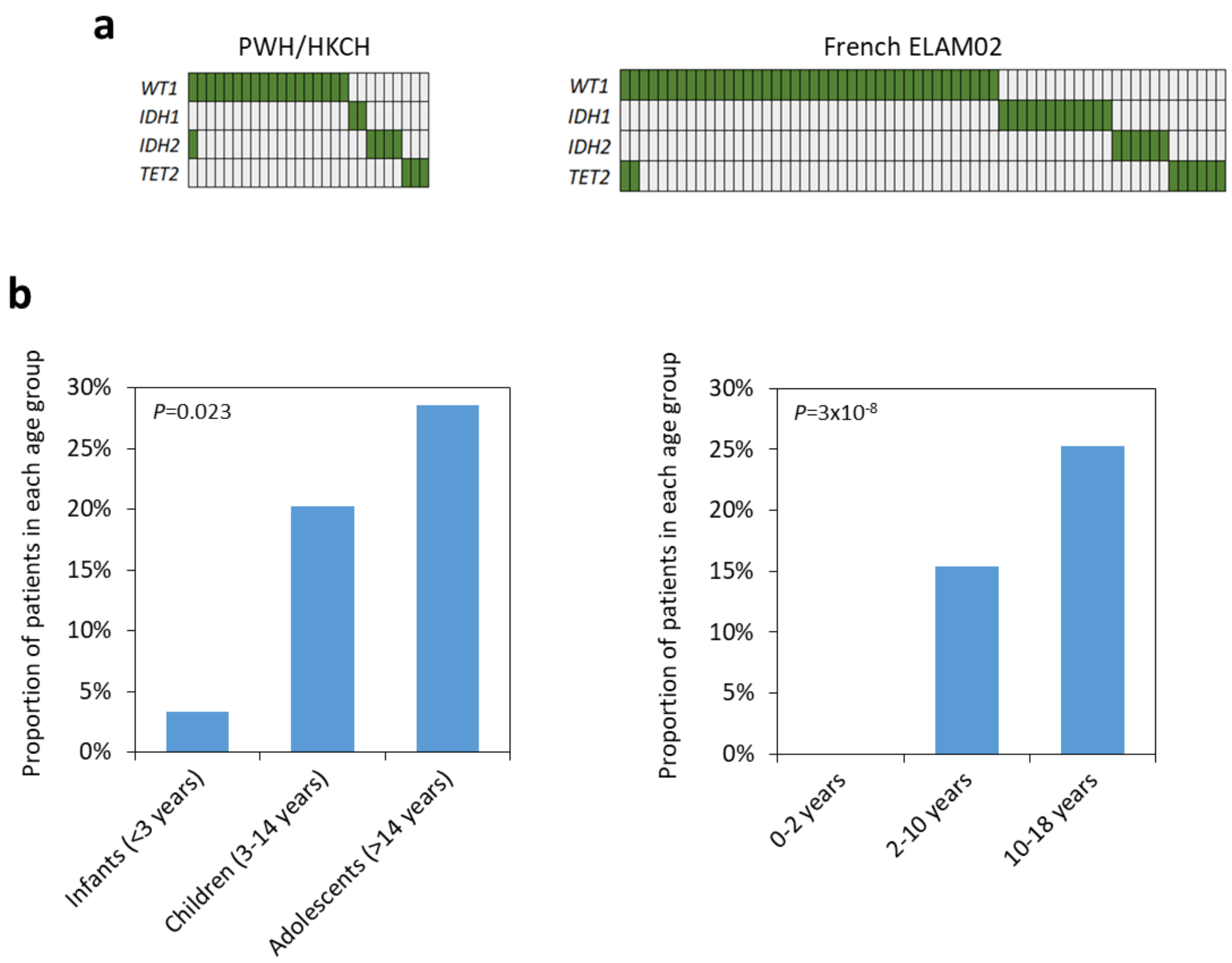

Supplementary Fig. 12. Mutations of the WIT pathway in pediatric AML. a Largely non-overlapping mutations of *WT1*, *IDH1/2* and *TET2* in the PWH/HKCH (*left*) and French ELAM02 (*right*) cohorts. **b** Age-related disruption of the WIT pathway in the two pediatric AML patient cohorts. *P*-values were calculated by the Fisher's exact test.

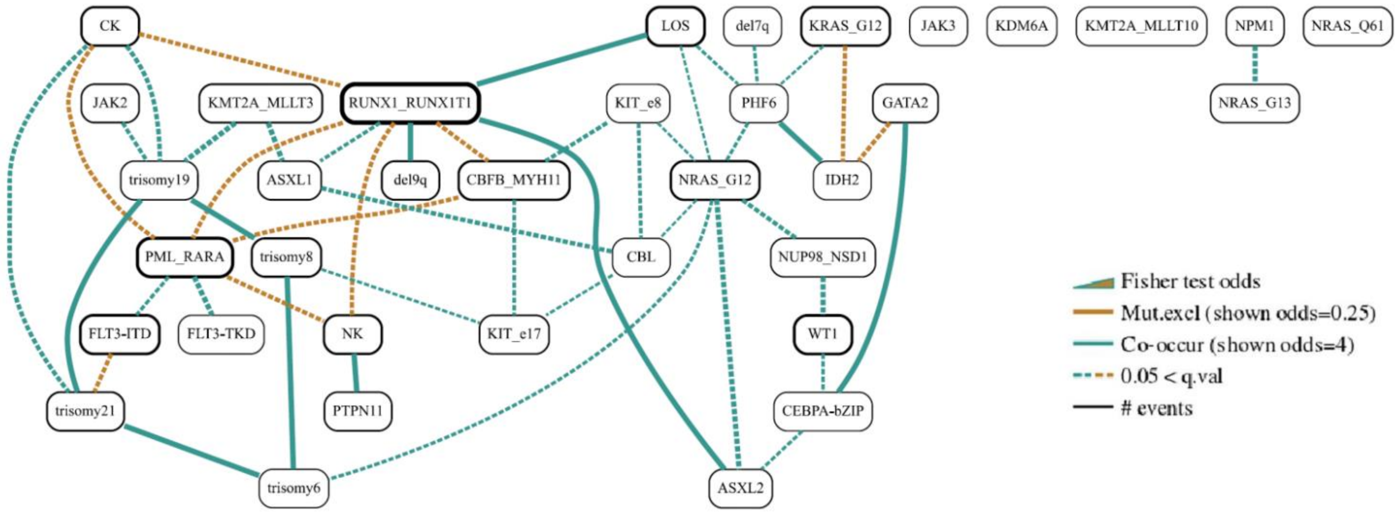

Supplementary Fig. 13. A Bayesian network showing the complex relationships among cytogenomic changes in pediatric AML patients. The network was constructed with the GOBNILP software program using the default settings (edge penalty=1).[3] Only those changes occurring in >3% of the entire cohort were included in the analysis. Edges visualised were based on Fisher's exact test corrected for multiple hypothesis testing. LOS, loss of sex chromosome.

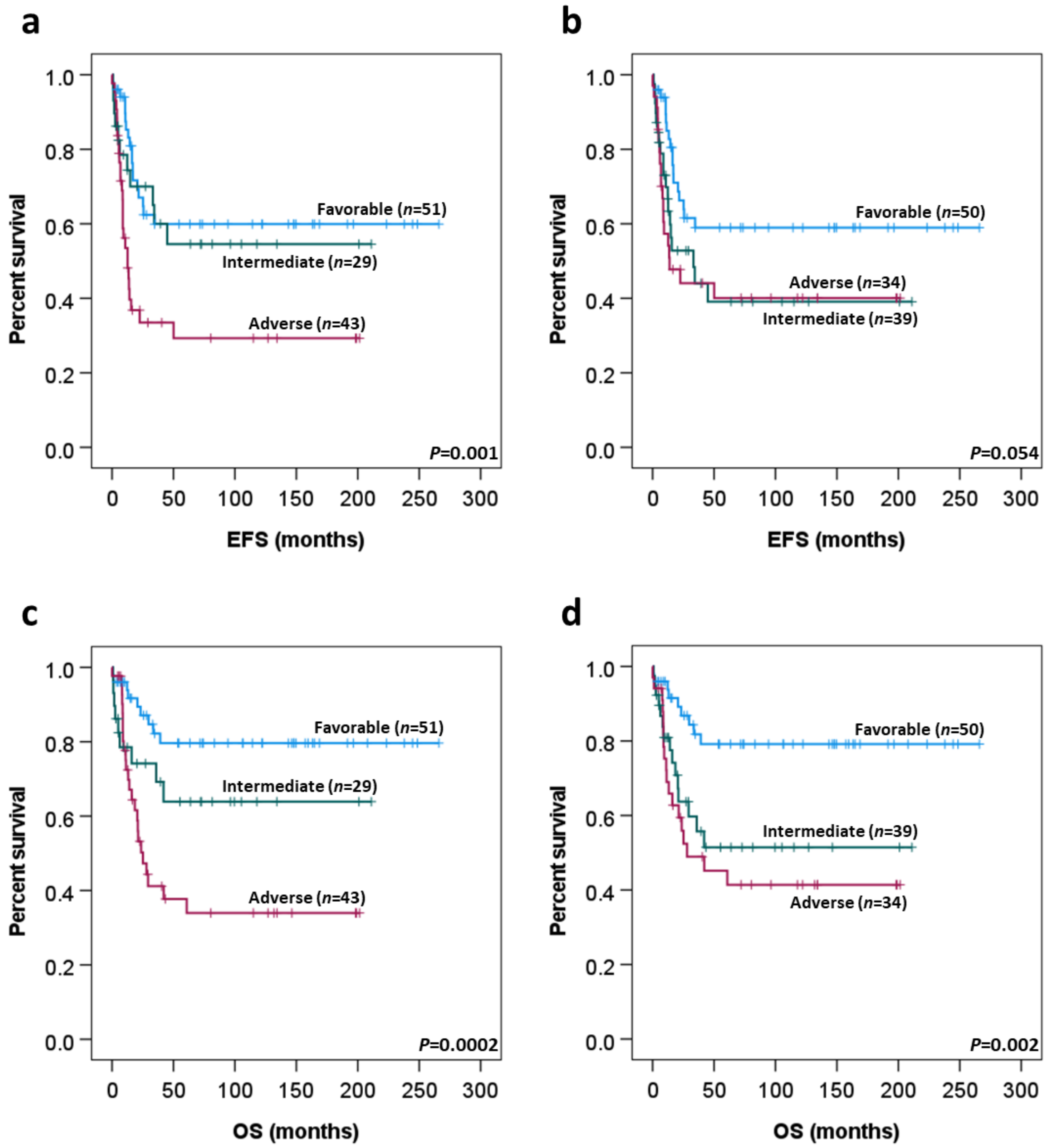

Supplementary Fig. 14. Prognostic risk stratification of pediatric AML patients. Kaplan-Meier analysis of EFS and OS based on the cytogenomic risk classification described in this study (**a**, **c**) or the 2022 ELN risk classification for adult AML patients (**b**, **d**).

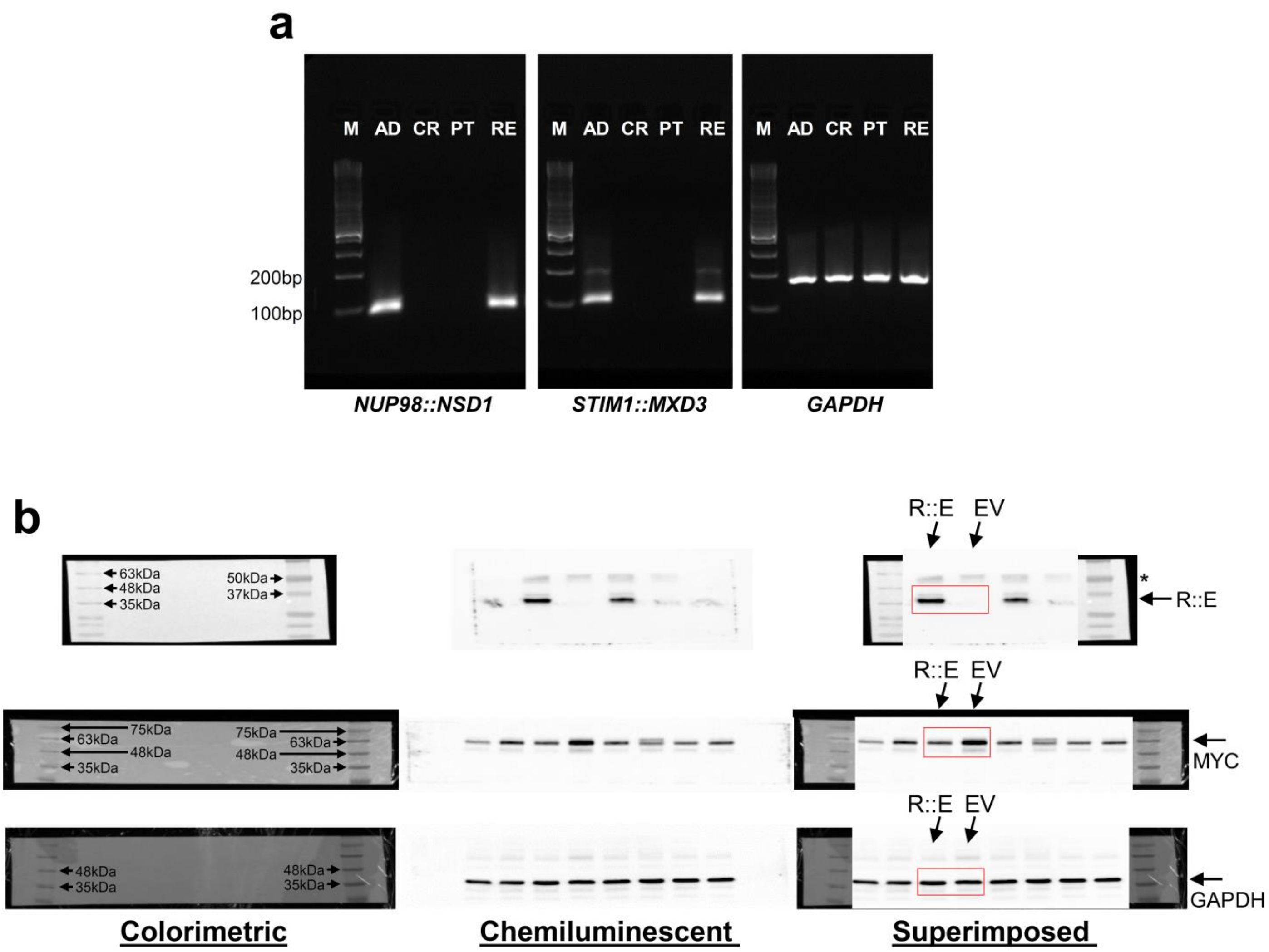

Supplementary Fig. 15. Uncropped blot and gel images. The uncropped versions of Fig. 1f (**a**) and Fig. 2f (**b**). For immunoblotting, two images were taken for each blot (colorimetric for ladder and chemiluminescent for bands) and the two images were superimposed in order to determine the molecular weight of the detected proteins. The asterisk in the R::E blot represents the endogenous RUNX1 that was also detected by the anti-AML1 (4334S, Cell Signaling Technology) antibody. Red boxes indicate the final cropped images shown in the main figure.

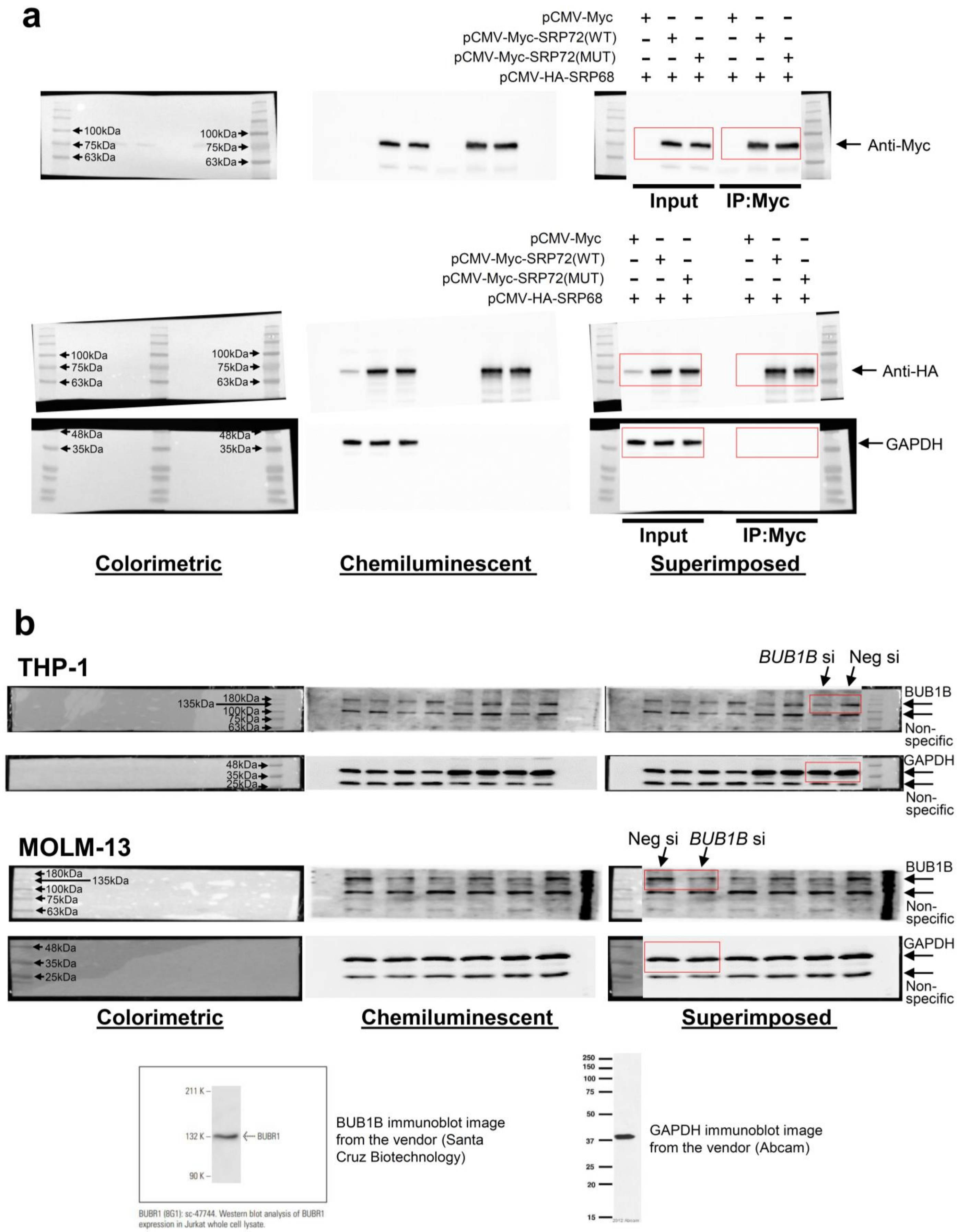

Supplementary Fig. 16. Uncropped blot images. The uncropped versions of Fig. 4e (**a**) and Fig. 6c (**b**). For immunoblotting, two images were taken for each blot (colorimetric for ladder and chemiluminescent for bands) and the two images were superimposed in order to determine the molecular weight of the detected proteins. Red boxes indicate the final cropped images shown in the main figure.

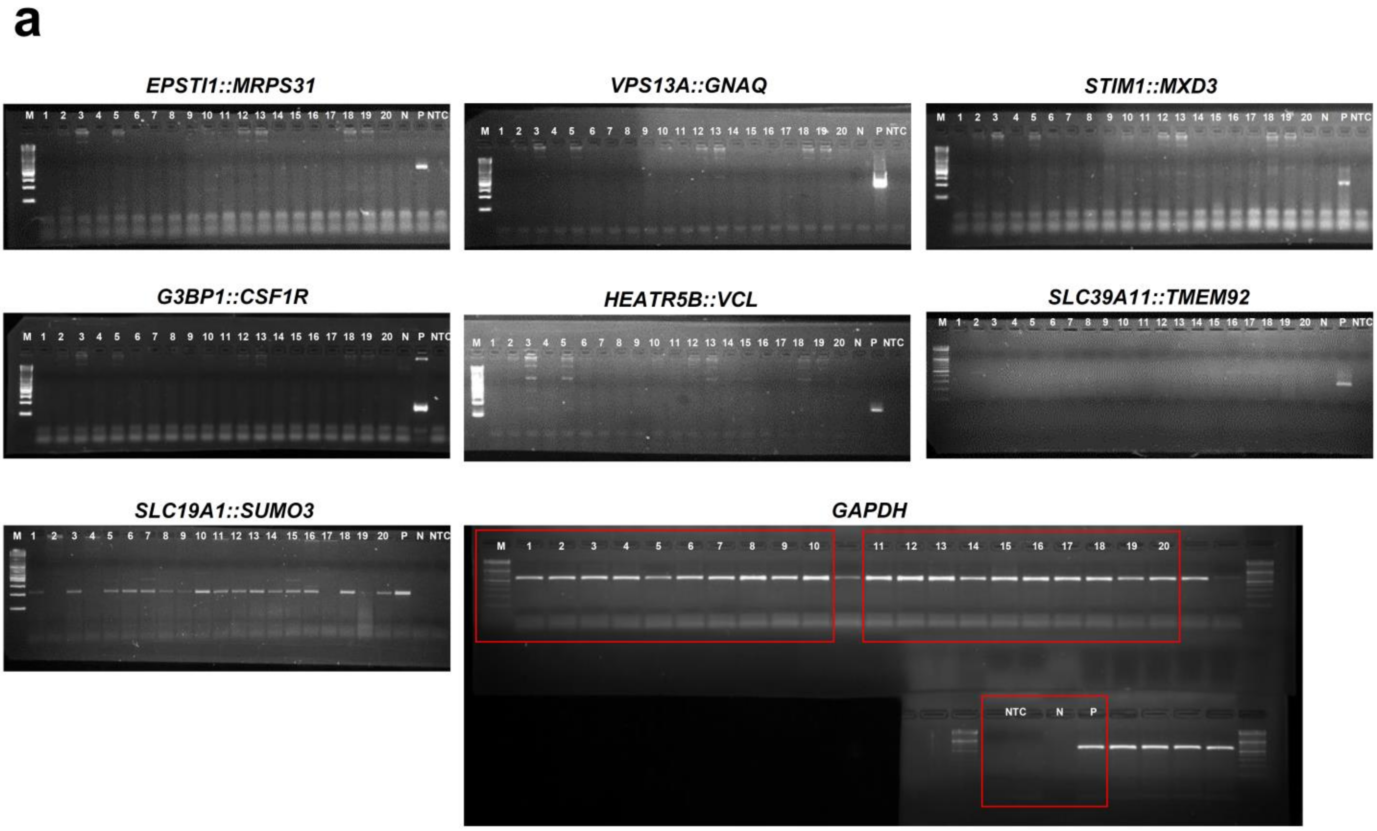

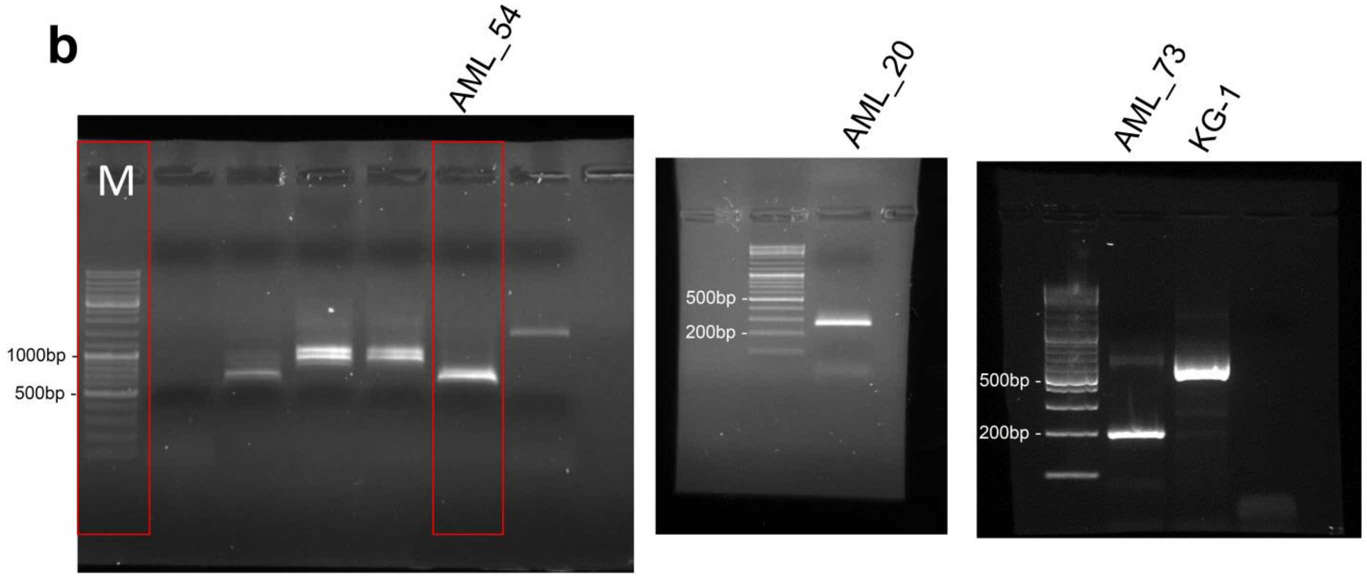

Supplementary Fig. 17. Uncropped gel images. The uncropped versions of Supplementary Fig. 3 (**a**) and Supplementary Fig. 9 (**b**). Red boxes indicate the merged lanes in the figure.

Supplementary Table 1. Characteristics and concurrent mutations in patients haboring novel FGs.

Sample	Novel FGs	Concurrent FGs	Concurrent mutated genes
AML_103	*RUNX1::ERG*	/	*SRP72*
AML_132	*G3BP1::CSF1R, VPS13A::GNAQ*	/	/
AML_96	*EPSTI1::MRPS31*	/	*WT1*
AML_50	*SLC39A11::TMEM92, SLC19A1::SUMO3*	/	*TP53*
AML_139	*STIM1::F12*	*NUP98::NSD1*	*PMS2, NTRK3*
AML_140	*STIM1::F12*	*NUP98::NSD1*	*WT1, NRAS, BCL6*
AML_123	*STIM1::MXD3*	*NUP98::NSD1*	*CEBPA, WT1, FLT3, P2RY2*
AML_81	*PHACTR4::COX10*	*NUP98::KDM5A*	/
AML_41	*HEATR5B::VCL*	*KMT2A::ARHGEF12*	*CUX1*
AML_145	*FMR1::BCOR*	*MN1::FLI1*	*CTCF, MLH1*

Supplementary Table 2. The 141 genes covered in the panel.

ABL1	*BRCA2*	*CTCF*	*FAS*	*JAK1*	*LUC7L2*	*NTRK3*	*PTPN11*	*SRP72*	*U2AF2*
ADA	*BRINP3*	*CUX1*	*FBXW7*	*JAK2*	*MAP2K1*	*OR13H1*	*RAD21*	*SRSF2*	*WAS*
ANKRD26	*C17orf97*	*DAXX*	*FLRT2*	*JAK3*	*MLH1*	*OR8B12*	*RB1*	*STAG2*	*WRN*
ASXL1	*CALR*	*DDX41*	*FLT3*	*KAT6A*	*MPL*	*P2RY2*	*RELN*	*STAT3*	*WT1*
ASXL2	*CARD11*	*DNM2*	*GATA1*	*KCNA4*	*MSH2*	*PAX5*	*RUNX1*	*STXBP2*	*XPO1*
ATM	*CBL*	*DNMT1*	*GATA2*	*KCNK13*	*MSH6*	*PCDHB1*	*SAXO2*	*SUZ12*	*ZRSR2*
ATRX	*CBLB*	*DNMT3A*	*GJB3*	*KDM6A*	*MYC*	*PDGFRA*	*SETBP1*	*TAL1*	
BCL6	*CBLC*	*EED*	*GNAS*	*KDR*	*MYD88*	*PHF6*	*SF1*	*TERC*	
BCOR	*CDKN2A*	*EGFR*	*HNRNPK*	*KIT*	*NBN*	*PML*	*SF3A1*	*TERT*	
BCORL1	*CEBPA*	*ELANE*	*HRAS*	*KLHDC8B*	*NF1*	*PMS2*	*SF3B1*	*TET2*	
BCR	*CHEK2*	*EP300*	*IDH1*	*KLHL6*	*NOTCH1*	*PRAMEF2*	*SH2B3*	*TNFRSF13B*	
BIRC3	*CREBBP*	*ETNK1*	*IDH2*	*KMT2A*	*NPAT*	*PRF1*	*SH2D1A*	*TP53*	
BLM	*CRLF2*	*ETV6*	*IKZF1*	*KMT2C*	*NPM1*	*PRPF40B*	*SMARCB1*	*TPMT*	
BRAF	*CSF1R*	*EZH2*	*IKZF3*	*KRAS*	*NRAS*	*PRPF8*	*SMC1A*	*TUBA3C*	
BRCA1	*CSF3R*	*FAM47A*	*IL7R*	*LRRC4*	*NSD1*	*PTEN*	*SMC3*	*U2AF1*	

Supplementary Table 3. Focal gene-level copy number alterations identified in the present cohort.

Sample	Focal copy number alterations	Genomic region	Validation
AML_74	*ABL1* loss	9q34.12	qPCR
AML_93	*BRCA1* loss	17q21.31	MLPA
AML_145	*BRINP3* gain	1q31.1	qPCR
AML_73	*CBL* loss (exons 8-9 deleted)	11q23.3	RT-PCR
AML_117	*CDKN2A* loss	9p21.3	MLPA
AML_20	*CEBPA* loss*	19q13.11	MLPA
AML_94	*CHEK2* loss	22q12.1	MLPA
AML_129	*CHEK2* loss (exons 5-15 deleted)*	22q12.1	MLPA
AML_129	*CREBBP* loss (exons 3-23 deleted)*	16p13.3	MLPA
AML_11	*CTCF* loss	16q22.1	qPCR
AML_57	*CTCF* loss	16q22.1	qPCR
AML_35	*ETV6* loss	12p13.2	qPCR
AML_74	*ETV6* loss	12p13.2	qPCR
AML_23	*KLHDC8B* loss	3p21.31	qPCR
AML_59	*KMT2A* loss (exons 9-36 deleted)	11q23.3	qPCR
AML_20	*KMT2A* -PTD	11q23.3	RT-PCR
AML_54	*KMT2A* -PTD	11q23.3	RT-PCR
AML_133	*KMT2A* -PTD	11q23.3	RT-PCR
AML_61	*MSH2* loss*	2p21	MLPA
AML_61	*MSH6* loss*	2p16.3	MLPA
AML_92	*MSH6* loss (exons 1-3 deleted)	2p16.3	MLPA
AML_74	*PHF6* loss	Xq26.2	qPCR
AML_92	*PMS2* loss	7p22.1	MLPA
AML_63	*PTPN11* loss	12q24.13	qPCR
AML_92	*PTPN11* loss	12q24.13	qPCR
AML_94	*PTPN11* loss	12q24.13	qPCR
AML_84	*SMC3* loss	10q25.2	qPCR
AML_115	*TET2* loss	4q24	qPCR
AML_94	*TP53* loss	17p13.1	MLPA
AML_50	*U2AF1* gain	21q22.3	qPCR
AML_3	*U2AF2* gain	19q13.42	qPCR
AML_48	*U2AF2* gain	19q13.42	qPCR
AML_74	*WT1* loss	11p13	qPCR

* Somatic as determined by remission blood sample testing.

Supplementary Table 4. Differential associations of pathway alterations with different molecular/cytogenetic subtypes in pediatric AML.

Functional pathways	R:R		C::M		P::R		*KMT2A* fusions		*NUP98* fusions		Rare fusions		*NPM1*		*CEBPA* -bZIP		NK		CK		Others		*P* -value*	Adjusted *P* -value#
	(total *n* =28)		(total *n* =13)		(total *n* =19)		(total *n* =20)		(total *n* =10)		(total *n* =10)		(total *n* =7)		(total *n* =5)		(total *n* =13)		(total *n* =13)		(total *n* =9)			
	n	%	*n*	%	*n*	%	*n*	%	*n*	%	*n*	%	*n*	%	*n*	%	*n*	%	*n*	%	*n*	%		
Transcription factor	2	7%	2	15%	1	5%	7	35%	4	40%	1	10%	2	29%	5	100%	7	54%	3	23%	3	33%	0.0001	**0.001**
Chromatin regulation	15	54%	0	0%	2	11%	9	45%	1	10%	2	20%	3	43%	1	20%	6	46%	2	15%	3	33%	0.003	**0.013**
Signaling	25	89%	13	100%	16	84%	14	70%	7	70%	4	40%	4	57%	3	60%	10	77%	6	46%	5	56%	0.004	**0.013**
Tumor suppressor	0	0%	0	0%	0	0%	3	15%	0	0%	0	0%	0	0%	0	0%	0	0%	4	31%	1	11%	0.007	**0.016**
DNA methylation	5	18%	3	23%	2	11%	0	0%	4	40%	1	10%	4	57%	2	40%	4	31%	1	8%	2	22%	0.016	**0.029**
Adhesion	2	7%	2	15%	1	5%	0	0%	0	0%	0	0%	0	0%	0	0%	0	0%	4	31%	0	0%	0.082	0.122
Splicing	1	4%	1	8%	0	0%	0	0%	0	0%	1	10%	1	14%	0	0%	1	8%	2	15%	2	22%	0.189	0.243
Cohesin	6	21%	2	15%	1	5%	1	5%	0	0%	2	20%	2	29%	0	0%	1	8%	2	15%	0	0%	0.430	0.484
DNA repair	1	4%	2	15%	1	5%	2	10%	2	20%	1	10%	1	14%	0	0%	2	15%	2	15%	3	33%	0.472	0.484

R::R, *RUNX1::RUNX1T1* ; C::M, *CBFB::MYH11* ; P::R, *PML::RARA* ; NK, normal karyotype; CK, complex karyotype.

* *P* -values were calculated by the Fisher's exact test.

# The Benjamini-Hochberg method was used for adjustment of multiple comparisons.

Supplementary Table 5. Associations of pathway alterations with age groups.

Functional pathways	Infants (<3 years) (total *n* =30)		Children (3-14 years) (total *n* =89)		Adolescents (>14 years) (total *n* =28)		*P* -value*	Adjusted *P* -value[#]
	n	**%**	***n***	**%**	***n***	**%**		
Epigenetic[≠]	4	13%	46	52%	17	61%	0.0001	**0.001**
Tumor suppressor	1	3%	1	1%	4	14%	0.009	**0.042**
Adhesion	3	10%	2	2%	4	14%	0.020	0.059
Signaling	19	63%	70	79%	18	64%	0.137	0.308
Transcription factor	4	13%	26	29%	7	25%	0.219	0.393
Cohesin	3	10%	8	9%	6	21%	0.221	0.618
NPM1	1	3%	3	3%	3	11%	0.248	0.618
Splicing	1	3%	5	6%	3	11%	0.549	0.618
DNA repair	3	10%	12	13%	2	7%	0.768	0.768

[≠] Chromatin regulation and DNA methylation were combined as epigenetic for the analysis.

* *P* -values were calculated by the Fisher's exact test.

[#] The Benjamini-Hochberg method was used for correction of multiple comparisons.

Supplementary Table 6. Comparison of cytogenomic changes in the PWH/HKCH and TARGET-AML cohorts.

**Cytogenomic alterations**	**PWH/HKCH cohort (≤18 years) (total *n* =140)**		**TARGET cohort (≤18 years) (total *n* =631)**		***P*- value***	**Adjusted *P*- value#**
	n	**%**	***n***	**%**		
FLT3	25	18%	208	33%	0.0003	**0.007**
Trisomy 21	10	7%	12	2%	0.003	**0.031**
NRAS	28	20%	195	31%	0.010	0.079
PHF6	6	4%	9	1%	0.039	0.232
KMT2A fusions	16	11%	116	18%	0.048	0.349
NPM1	7	5%	65	10%	0.054	0.349
-Y	12	9%	31	5%	0.102	0.349
del(9q)	10	7%	26	4%	0.125	0.375
ASXL1	9	6%	23	4%	0.157	0.418
CBFB::MYH11	13	9%	89	14%	0.167	0.484
TET2	3	2%	32	5%	0.177	0.484
ASXL2	8	6%	23	4%	0.242	0.484
RUNX1::RUNX1T1	28	20%	100	16%	0.258	0.491
PTPN11	7	5%	51	8%	0.287	0.491
del(7q)	5	4%	13	2%	0.347	0.555
CEBPA	7	5%	48	8%	0.364	0.608
CBL	6	4%	19	3%	0.431	0.608
GATA2	8	6%	29	5%	0.519	0.691
KIT	15	11%	81	13%	0.572	0.723
KRAS	15	11%	81	13%	0.572	0.906
IDH2	5	4%	17	3%	0.575	0.906
WT1	16	11%	86	14%	0.582	0.906
Trisomy 8	11	8%	54	9%	0.868	0.906
-X	5	4%	22	3%	1.000	1.000

* *P* -values were calculated by the Fisher's exact test.

# The Benjamini-Hochberg method was used for correction of multiple comparisons.

Supplementary Table 7. Baseline characteristics of the patients treated with the three chemotherapy protocols.

Variables	Modified UK MRC AML 12 (*n* =46)	%	NOPHO-AML 2004 (*n* =43)	%	NOPHO-DBH AML 2012 (*n* =34)	%	*P* -value*
Male sex, *n*	29	63%	26	60%	20	59%	0.946
Age, median (range), years	9.8 (0.7-17)	/	10 (0.3-17)	/	9.4 (0.2-17)	/	0.814
WBC, median (range), x10^9/L	25.3 (1.4-352)	/	18.7 (0.7-263.1)	/	23.3 (1.6-218.6)	/	0.437
Cytogenomic risk groups							0.163
Adverse	12	26%	15	35%	16	47%	
Non-adverse	34	74%	28	65%	18	53%	

WBC, presentation white blood cell counts.

* Categorical and continuous variables were analysed by the Fisher's exact and Kruskal-Wallis test, respectively.

Supplementary Table 8. Univariate analysis of EFS and OS.

Variables[#]	*n*	EFS			OS		
		HR	95% CI	*P*-value	HR	95% CI	*P*-value
Male sex	75	0.712	0.421-1.204	0.205	0.603	0.329-1.105	0.102
Age	/	0.997	0.951-1.045	0.898	0.998	0.944-1.055	0.944
Presentation WBC counts	/	1.004	1.000-1.007	**0.035**	1.002	0.997-1.006	0.437
Adverse cytogenomic risk*	43	2.557	1.505-4.347	**0.001**	3.202	1.728-5.934	**0.0002**
NOPHO-AML 2004	43	1.336	0.739-2.414	0.338	1.553	0.772-3.123	0.217
NOPHO-DBH-AML 2012	34	1.346	0.654-2.774	0.42	1.968	0.854-4.537	0.112
CR after first induction course	92	0.482	0.275-0.847	**0.011**	0.321	0.172-0.596	**0.0003**
SCT at CR1	21	0.564	0.255-1.246	0.157	0.904	0.401-2.037	0.808
Number of mutations	/	0.867	0.723-1.039	0.121	0.897	0.732-1.100	0.296
Genes / distinct mutation types							
NRAS	27	0.937	0.495-1.774	0.841	0.918	0.439-1.920	0.821
KRAS	14	0.594	0.237-1.491	0.267	0.296	0.071-1.224	0.093
PTPN11	9	0.415	0.101-1.702	0.222	0.629	0.152-2.604	0.522
JAK2	9	0.913	0.285-2.923	0.878	1.315	0.406-4.262	0.648
GATA2	9	1.274	0.508-3.196	0.605	1.811	0.711-4.610	0.213
ASXL1	9	0.735	0.230-2.352	0.604	0.315	0.043-2.292	0.254
KIT-ex17	8	0.939	0.339-2.597	0.903	0.638	0.154-2.642	0.535
ASXL2	8	0.671	0.210-2.147	0.501	0.599	0.145-2.481	0.48
CBL[≠]	6	1.029	0.321-3.297	0.961	0.422	0.058-3.069	0.394
PHF6[≠]	6	0.235	0.033-1.703	0.152	0.362	0.050-2.635	0.316
KDM6A	6	0.967	0.302-3.098	0.955	0.433	0.060-3.150	0.408
NPM1	6	0.044	0.001-3.841	0.171	0.045	0.000-10.045	0.261
CEBPA-bZIP	5	0.685	0.167-2.810	0.599	0.949	0.229-3.928	0.942
KIT-ex8	5	0.54	0.075-3.905	0.541	0.047	0.000-75.569	0.417
JAK3	5	1.263	0.307-5.187	0.746	1.915	0.461-7.951	0.371
IDH2	5	0.782	0.190-3.211	0.732	0.557	0.077-4.048	0.563
DNM2	4	1.411	0.440-4.520	0.562	0.557	0.077-4.054	0.564
CEBPA[≠]	5	1.801	0.562-5.769	0.322	2.417	0.742-7.874	0.143
KMT2C	4	1.372	0.333-5.653	0.661	0.703	0.097-5.124	0.728
TP53[≠]	6	6.392	2.703-15.118	**2.4×10^{-5}**	8.351	3.442-20.261	**2.7×10^{-6}**
TP53 mutation	3	12.056	3.509-41.421	**7.7×10^{-5}**	15.427	4.359-54.601	**2.2×10^{-5}**
TP53 deletion	3	4.079	1.269-13.115	**0.018**	5.162	1.584-16.825	**0.006**

WBC, white blood cell; CR, complete remission; SCT, stem cell transplantation; *CEBPA* sm, *CEBPA* single mutation; EFS, event-free survival; OS, overall survival; HR, hazard ratios; 95% CI, 95% confidence interval.

[#] Age, presentation WBC counts and the number of mutations were analysed as continuous variables. The modified UK MRC AML 12 protocol was used as the reference when compared to other protocols.

* Includes complex karyotype, -7, -5, del(5q), del(12p), *WT1, FLT3*-ITD, *DEK::NUP214*, *KMT2A::AFDN*, *KMT2A::MLLT10*, *NUP98* fusions, *FUS::ERG* and *CBF2AT3::GLIS2*.

[≠] Mutations and deletions of these genes were considered together as the changes could be categorised as functional loss.

Supplementary Table 9. Characteristics of pediatric AML patients with *TP53* alterations.

Sample	*TP53*	Sex	Age	WBC, x10^{9}/L	FAB subtypes	Cytogenetics	Molecular/cytogenetic subtypes	Ethnicity
AML_105	mutation	M	17	6.3	M7	47,XY,+1,-13,+21[2]/46,idem,del(Y)(q11.23q12),add(3)(q25),-4,-14,+mar1[9]/46,idem,del(Y)(q11.23q12),ins(2;?)(p13;?),add(3)(q25),-4,-14,+mar2[8]/46,XY[1]	Complex karyotype	Chinese
AML_127	mutation	M	16	164.2	M5	46,X,-Y,-13,+marx2[18]	Complex karyotype	Chinese
AML_50	mutation	M	16	0.7	M0	44,XY,del(1)(p11),-4,del(5)(q13q31),-7,der(14;21)(q10;q10),+mar[20]	Complex karyotype	Chinese
AML_108	deletion	F	16	35.7	M5	46,XX,t(9;11)(p22;q23)[14]/46,idem,i(17)(q10)[6]	*KMT2A* fusions	Chinese
AML_142	deletion	M	16	34.6	M1	44~48,XY,add(3)(p21)[6],add(3)(q12)[4],-5[5],add(5)(p15)[2],add(5)(q13)[7],add(6)(q13)[8],-7[11],add(9)(p13)[9],-14[3],-17[9],+1~6mar[cp13]/45,XY,add(3)(p21),add(5)(q13),add(6)(q13),-7,add(9)(p13),-17,+mar[5]/46,XY[1]	Complex karyotype	Chinese
AML_94	deletion	F	1	5.8	Unknown	46,XX,t(5;6)(q33;q33)[22]	Others	Chinese

WBC, presentation white blood cell counts; FAB, French-American-British.

Supplementary Table 10. The 56 pediatric AML patients with transcriptome sequencing done for *TP53* pathway analysis.

**Sample**	***TP53* status**	**Molecular/cytogenetic subtypes**
AML_105	altered	Complex karyotype
AML_108	altered	*KMT2A* fusions
AML_127	altered	Complex karyotype
AML_142	altered	Complex karyotype
AML_50	altered	Complex karyotype
AML_94	altered	Others
AML_100	wt	*RUNX1::RUNX1T1*
AML_102	wt	*CBFB::MYH11*
AML_103	wt	Others
AML_104	wt	*RUNX1::RUNX1T1*
AML_106	wt	*RUNX1::RUNX1T1*
AML_107	wt	Rare fusions
AML_111	wt	*NUP98* fusions
AML_112	wt	*CBFB::MYH11*
AML_113	wt	*RUNX1::RUNX1T1*
AML_114	wt	Complex karyotype
AML_115	wt	*RUNX1::RUNX1T1*
AML_116	wt	*RUNX1::RUNX1T1*
AML_117	wt	*KMT2A* fusions
AML_118	wt	Normal karyotype
AML_121	wt	Others
AML_123	wt	*NUP98* fusions
AML_124	wt	*KMT2A* fusions
AML_125	wt	*RUNX1::RUNX1T1*
AML_126	wt	*RUNX1::RUNX1T1*
AML_129	wt	*KMT2A* fusions
AML_132	wt	Complex karyotype
AML_133	wt	Normal karyotype
AML_135	wt	*KMT2A* fusions
AML_136	wt	Others
AML_137	wt	*NPM1*
AML_139	wt	*NUP98* fusions
AML_140	wt	*NUP98* fusions
AML_143	wt	*KMT2A* fusions
AML_144	wt	Rare fusions
AML_145	wt	Complex karyotype
AML_146	wt	*RUNX1::RUNX1T1*
AML_147	wt	*RUNX1::RUNX1T1*
AML_148	wt	*KMT2A* fusions
AML_20	wt	Normal karyotype
AML_27	wt	*NPM1*
AML_35	wt	Complex karyotype
AML_41	wt	*KMT2A* fusions
AML_44	wt	Complex karyotype
AML_52	wt	Normal karyotype
AML_55	wt	*NUP98* fusions
AML_69	wt	*RUNX1::RUNX1T1*
AML_70	wt	Normal karyotype
AML_71	wt	Normal karyotype
AML_79	wt	Others
AML_81	wt	*NUP98* fusions
AML_92	wt	Normal karyotype
AML_95	wt	*RUNX1::RUNX1T1*
AML_96	wt	Normal karyotype
AML_98	wt	*CEBPA* -bZIP
AML_99	wt	*NUP98* fusions

Supplementary Table 11. Characteristics of the PWH/HKCH, SCMC and TARGET pediatric AML cohorts.[#]

	PWH/HKCH cohort (*n* =140)		SCMC cohort (*n* =292)		TARGET cohort (*n* =631)		*P* -value*		
							PWH/HKCH vs. SCMC	PWH/HKCH vs. TARGET	SCMC vs. TARGET
**Platform for mutation detection**	**DNA-based**		**RNA-based**		**DNA-based**				
	n	**%**	***n***	**%**	***n***	**%**			
**Sex**							0.675	0.024	0.016
Female	53	38%	117	40%	307	49%			
Male	87	62%	175	60%	324	51%			
**FAB subtypes**							$4x10^{-6}$	0.001	$1.1x10^{-20}$
M0/M1	20	14%	10	3%	91	14%			
M2	33	24%	110	38%	146	23%			
M4	23	16%	34	12%	148	23%			
M5	26	19%	95	33%	112	18%			
M7	14	10%	40	14%	15	2%			
**Age groups**							$3x10^{-12}$	0.92	$8.1x10^{-18}$
<3	28	20%	107	37%	118	19%			
3-15	84	60%	181	62%	380	60%			
15-18	28	20%	4	1%	133	21%			

[#] Data of the SCMC and TARGET cohorts were obtained from Liu *et al* .[35] Only Chinese patients in the PWH/HKCH cohort (140 of 147 patients) were included in the comparison.

* Fisher's exact test was used to compare sex, while chi-square test was used to compare FAB and age group distribution.

Supplementary References

1. Bolouri, H., et al. The molecular landscape of pediatric acute myeloid leukemia reveals recurrent structural alterations and age-specific mutational interactions. *Nat. Med*. **24**, 103-112 (2018).
2. Zhou, X., et al. Exploring genomic alteration in pediatric cancer using ProteinPaint. *Nat. Genet.* **48**, 4-6 (2016).
3. Bartlett, M. & Cussens, J. Integer linear programming for the Bayesian network structure learning problem. *Artif. Intell*. **244**, 258-271 (2017).
