# Supplementary material for: Deep genomic characterization highlights complexities and prognostic markers of pediatric acute myeloid leukemia
Source: Commun Biol. 2023 Mar 31;6:356. doi: 10.1038/s42003-023-04732-2 (PMC10066286; doi:10.1038/s42003-023-04732-2)
Supplement: Supplementary file 3 — Description of Additional Supplementary Files [file 42003_2023_4732_MOESM3_ESM.pdf]

Description of Additional Supplementary Files

File name: Supplementary Data 1

Description: Characteristics of the pediatric AML patient cohort.

File name: Supplementary Data 2

Description: The complete list of the 336 mutations.

File name: Supplementary Data 3

Description: Differential gene expression analysis of *TP53*-altered vs. *TP53*-wild-type pediatric AML patients by DESeq2.

File name: Supplementary Data 4

Description: Gene effect scores in *TP53*-altered and *TP53*-wild-type AML cell lines from the DepMap datasets.

File name: Supplementary Data 5

Description: Primer list.

File name: Supplementary Data 6

Description: The source data behind the graphs and charts in the paper.
