# Supplementary material for: Deep genomic characterization highlights complexities and prognostic markers of pediatric acute myeloid leukemia
Source: Commun Biol. 2023 Mar 31;6:356. doi: 10.1038/s42003-023-04732-2 (PMC10066286; doi:10.1038/s42003-023-04732-2)
Supplement: Supplementary file 10 — Reporting Summary [file 42003_2023_4732_MOESM10_ESM.pdf]

nature portfolio

Corresponding author(s): Margaret HL Ng

Last updated by author(s): Mar 8, 2023

Reporting Summary

Nature Portfolio wishes to improve the reproducibility of the work that we publish. This form provides structure for consistency and transparency in reporting. For further information on Nature Portfolio policies, see our Editorial Policies and the Editorial Policy Checklist.

Statistics

For all statistical analyses, confirm that the following items are present in the figure legend, table legend, main text, or Methods section.

n/a	Confirmed	
☐	☒	The exact sample size (*n*) for each experimental group/condition, given as a discrete number and unit of measurement
☐	☒	A statement on whether measurements were taken from distinct samples or whether the same sample was measured repeatedly
☐	☒	The statistical test(s) used AND whether they are one- or two-sided<br>*Only common tests should be described solely by name; describe more complex techniques in the Methods section.*
☐	☒	A description of all covariates tested
☐	☒	A description of any assumptions or corrections, such as tests of normality and adjustment for multiple comparisons
☐	☒	A full description of the statistical parameters including central tendency (e.g. means) or other basic estimates (e.g. regression coefficient) AND variation (e.g. standard deviation) or associated estimates of uncertainty (e.g. confidence intervals)
☐	☒	For null hypothesis testing, the test statistic (e.g. *F*, *t*, *r*) with confidence intervals, effect sizes, degrees of freedom and *P* value noted<br>*Give P values as exact values whenever suitable.*
☒	☐	For Bayesian analysis, information on the choice of priors and Markov chain Monte Carlo settings
☒	☐	For hierarchical and complex designs, identification of the appropriate level for tests and full reporting of outcomes
☒	☐	Estimates of effect sizes (e.g. Cohen's *d*, Pearson's *r*), indicating how they were calculated

Our web collection on statistics for biologists contains articles on many of the points above.

Software and code

Policy information about availability of computer code

Data collection	No software was used.
Data analysis	SPSS Statistics 27 was used.

For manuscripts utilizing custom algorithms or software that are central to the research but not yet described in published literature, software must be made available to editors and reviewers. We strongly encourage code deposition in a community repository (e.g. GitHub). See the Nature Portfolio guidelines for submitting code & software for further information.

Data

Policy information about availability of data

All manuscripts must include a data availability statement. This statement should provide the following information, where applicable:

- Accession codes, unique identifiers, or web links for publicly available datasets
- A description of any restrictions on data availability
- For clinical datasets or third party data, please ensure that the statement adheres to our policy

Raw data for targeted myeloid sequencing and RNA-seq can be found in NCBI Sequence Read Archive (PRJNA924067 and PRJNA924068) and Gene Expression Omnibus (accession number GSE222903). Source data underlying graphs and charts are provided in Supplementary Data 6. Uncropped blot and gel images are provided in Supplementary Fig. 15-17. All other data and research materials are available from the corresponding author on reasonable request.

Human research participants

Policy information about studies involving human research participants and Sex and Gender in Research.

Reporting on sex and gender	147 pediatric AML patients including 91 males and 56 females were studied. The relationships of sex with genetic/ cytogenetic information and survivals were analysed.
Population characteristics	Age, laboratory findings (e.g. presentation white blood cell counts and disease subtypes), treatment categories, genetic and cytogenetic information.
Recruitment	Patients (<=18 years old) with newly diagnosed acute myeloid leukemia were recruited in Prince of Wales Hospital (PWH) and Hong Kong Children's Hospital (HKCH).
Ethics oversight	The Joint Chinese University of Hong Kong-New Territories East Cluster Clinical Research Ethics Committee and Hong Kong Children's Hospital Research Ethics Committee.

Note that full information on the approval of the study protocol must also be provided in the manuscript.

Field-specific reporting

Please select the one below that is the best fit for your research. If you are not sure, read the appropriate sections before making your selection.

☒ Life sciences ☐ Behavioural & social sciences ☐ Ecological, evolutionary & environmental sciences

For a reference copy of the document with all sections, see nature.com/documents/nr-reporting-summary-flat.pdf

Life sciences study design

All studies must disclose on these points even when the disclosure is negative.

Sample size	No sample-size calculation was performed in this study. As pediatric AML is a rare disease, we have been collecting patient samples from August 1997 to January 2022 to reach a sample size of 147, which is comparable to the size of the discovery cohort (n=197) in the Children's Oncology Group (COG)-National Cancer Institute (NCI) TARGET-AML study.
Data exclusions	Secondary/therapy-related AML and Down syndrome cases were excluded.
Replication	As pediatric AML is a rare disease, we could not recruit another independent cohort to validate the findings in this study. However, we did compare our findings with those reported in other studies (e.g. the TARGET-AML and French ELAM02 studies) to evaluate the reproducibility of the observed results. In this regard, we could consistently observe predominant signaling gene mutations, higher incidence of DNA repair alterations and age-related disruption of the WIT pathway in pediatric AML patients. However, we failed to observe the dramatic differences in the genomic landscape between Chinese and Western pediatric AML patients as previously reported. This discrepancy could be attributed to the considerable differences in the characteristics of the study cohorts and the different platforms used in mutation detection that might have led to varied frequencies in particularly tumor suppressor genes.<br>Regarding cell line studies, all experiments were performed independently for 2-4 times (many of the them with replicates performed in each experiment) to verify the findings. For RUNX1::ERG, we tested the fusion gene impacts in two different myeloid cell lines and primary CD34+ hematopoietic stem/progenitor cells with a variety of cell-based assays and similar conclusions could be drawn from the findings. Likewise, for TP53 editing, two CRISPR clones with different genotypes were analysed and consistent effects could be observed.
Randomization	Not relevant as this study is not a clinical trial study.
Blinding	Not relevant as this study is not a clinical trial study.

Reporting for specific materials, systems and methods

We require information from authors about some types of materials, experimental systems and methods used in many studies. Here, indicate whether each material, system or method listed is relevant to your study. If you are not sure if a list item applies to your research, read the appropriate section before selecting a response.

Materials & experimental systems

n/a	Involved in the study
	☒ Antibodies
	☒ Eukaryotic cell lines
☒	Palaeontology and archaeology
☒	Animals and other organisms
	☒ Clinical data
☒	Dual use research of concern

Methods

n/a	Involved in the study
☒	ChIP-seq
	☒ Flow cytometry
☒	MRI-based neuroimaging

Antibodies

Antibodies used

Anti-c-Myc (supplier: Invitrogen, catalog no.: MA1-980, clone name: 9E10, lot no.: WJ332704, concentration: 0.5 mg/mL, purpose: IP), anti-HA-Tag (supplier: Cell Signaling Technology, catalog no.: 3724S, clone name: C29F4, lot no.: 10, purpose: immunoblotting/IF), anti-Myc-Tag (supplier: Cell Signaling Technology, catalog no.: 2276S, clone name: 9B11, lot no.: 24, purpose: immunoblotting/IF), anti-AML1 (supplier: Cell Signaling Technology, catalog no.: 4334S, lot no.: 3, purpose: immunoblotting), anti-c-Myc (supplier: Cell Signaling Technology, catalog no.: 5605S, clone name: D84C12, lot no.: 2, purpose: immunoblotting), anti-BUBR1 (supplier: Santa Cruz Biotechnology, catalog no.: sc-47744, clone name: 8G1, lot no.: D2021, concentration: 200 µg/mL, purpose: immunoblotting), anti-GAPDH (supplier: Abcam, catalog no.: ab9485, lot no.: GR3391612-1, purpose: immunoblotting), goat anti-mouse IgG (H+L) cross-adsorbed secondary antibody, Alexa Fluo 488 (supplier: Invitrogen, catalog no.: A11001, lot no.: 1726530, purpose: IF), donkey anti-rabbit IgG (H+L) highly cross-adsorbed secondary antibody, Alexa Fluor 488 (supplier: Invitrogen, catalog no.: A21206, lot no.: 1608521, purpose: IF), goat anti-rabbit immunoglobulins/HRP (supplier: Dako, catalog no.: P0448, lot no.: 41255488A, purpose: immunoblotting), goat anti-mouse immunoglobulins/HRP (supplier: Dako, catalog no.: P0447, lot no.: 20062696, purpose: immunoblotting).

Validation

According to the manufacturer's website (Cell Signaling Technology), anti-c-Myc (D84C12) is intended for use in Western blotting to detect endogenous levels of total c-Myc protein and has been cited 757 times. Also, the AML1 antibody (4334S), raised against the amino terminus of human RUNX1, is intended for Western blotting to detect RUNX1 and the RUNX1::RUNX1T1 fusion protein. It has been referenced in 42 publications. Also, this antibody has been employed in the study of RUNX1::RUNX1T1 in an article recently published in Nature Communications (doi: 10.1038/s41467-020-20848-z.). For anti-BUBR1 (sc-47744), this antibody is recommended for Western blotting and has been cited 7 times according to the manufacturer's website (Santa Cruz Biotechnology). Consistent BUB1B knockdown results by qPCR and Western blotting indicated the validity of the antibody. The loading control anti-GAPDH (ab9485) as well as the anti-HA-Tag (C29F4) and anti-Myc-Tag (9B11) antibodies for detection of tagged proteins have been widely used and referenced in more than 1,500 publications according to the manufacturer's websites.

Eukaryotic cell lines

Policy information about cell lines and Sex and Gender in Research

Cell line source(s)

HeLa, 293T and K562 were obtained from ATCC, while U937, THP-1 and MOLM-13 were purchased from DSMZ.

Authentication

All cell lines were verified by short tandem repeat analysis using the CLA IdentiFiler Plus PCR Amplification Kit (Thermo Fisher Scientific).

Mycoplasma contamination

The cell lines were not tested for mycoplasma contamination.

Commonly misidentified lines
(See ICLAC register)

Nil.

Clinical data

Policy information about clinical studies

All manuscripts should comply with the ICMJE guidelines for publication of clinical research and a completed CONSORT checklist must be included with all submissions.

Clinical trial registration

This study is not a clinical trial study.

Study protocol

This study is not a clinical trial study.

Data collection

The 147 pediatric AML cases were diagnosed between August 1997 and January 2022. Clinical data including patient demographics, laboratory findings, the date of initial diagnosis, family history, the treatment regimen received, treatment response, the date of complete remission (if any), the date of relapse (if any), the date of stem cell transplantation (if any), the date of death (if any) and the date of last follow-up were periodically retrieved from electronic patient records from the hospital information system for survival studies. Data were last updated in April 2022.

Outcomes

Event-free survival (EFS) was measured from the date of diagnosis until failure to achieve complete remission, relapse, or death from any cause (whichever occurred first), censoring for those alive and event-free at last follow-up. Overall survival (OS) was measured from the date of diagnosis until death from any cause, censoring for those alive at last follow-up. Univariate cox regression analysis was used to evaluate the significance of each parameter on survivals. Variables with P<0.05 in univariate Cox regression analysis were

included in multivariate analysis with adjustment for other potential confounding variables including treatment protocols and stem cell transplantation.

Flow Cytometry

Plots

Confirm that:

☒ The axis labels state the marker and fluorochrome used (e.g. CD4-FITC).

☒ The axis scales are clearly visible. Include numbers along axes only for bottom left plot of group (a 'group' is an analysis of identical markers).

☒ All plots are contour plots with outliers or pseudocolor plots.

☒ A numerical value for number of cells or percentage (with statistics) is provided.

Methodology

Sample preparation	For DNA content (cell cycle and apoptosis) analysis, cells were fixed in pre-chilled 70% ethanol, washed twice with 1 × PBS, and incubated with the PI/RNase Staining Buffer (BD Biosciences) for 15 minutes at room temperature before analysis. For other functional assays such as evaluating proliferation and protein synthesis, the sample preparation steps as described in the manufacturer's protocols were followed.
Instrument	BD FACSCalibur flow cytometer (BD Biosciences).
Software	FlowJo v7.6.5 was used for flow cytometry data analysis.
Cell population abundance	CD34+ hematopoietic stem/progenitor cells were isolated from cord blood samples using the Indirect CD34 MicroBead Kit from Miltenyi Biotec. The purity of the isolated cells was evaluated by flow cytometric analysis of CD34 expression, which was consistently over 90% in 3 separate experiments. Also, we performed CD34+ cell sorting for the remission bone marrow samples from the patient case AML_103 carrying the RUNX1::ERG fusion. The purity of CD34+ cells was found to be at least 90%. The purified CD34+ cells were used for transcriptome sequencing as described in the text.<br>Regarding nucleofection of K562 cells, our established protocol consistently yielded a transfection efficiency over 70%, which is similar to that reported in the manufacturer's website. For lentiviral transduction of U937 cells, the efficiency was around 60-90%. For lentiviral transduction of primary CD34+ hematopoietic stem/progenitor cells, the efficiency was around 10%. These values were determined by flow cytometric analysis of green fluorescent protein (GFP), which was co-expressed by the LeGO-iG2 vectors used in the experiments. As the transduction efficiency in CD34+ hematopoietic stem/progenitor cells was low, transduced cells (GFP-positive) were purified by fluorescence-activated cell sorting using the BD FACSAria Fusion cell sorter before colony-forming unit (CFU) analysis.
Gating strategy	For functional assays, cells were first gated with FSC/SSC to identify the target cell population while excluding debris in the density plot. As the expression vectors used in these experiments co-expressed green fluorescent protein (GFP), cells were then gated to identify GFP-positive cells. The boundaries between "positive" and "negative" were defined by <1% of GFP positivity in untransfected/untransduced cells in histogram plots. After that, the GFP-positive (transfected/transduced) cells were analysed for the desired functional properties. For cell cycle analysis, doublet exclusion using a FL2-W vs. FL2-A gating was performed to ensure analysis of single cells.

☒ Tick this box to confirm that a figure exemplifying the gating strategy is provided in the Supplementary Information.
